# Leaky‐Integrate‐Fire Neuron via Synthetic Antiferromagnetic Coupling and Spin‐Orbit Torque

**DOI:** 10.1002/advs.202521732

**Published:** 2026-02-12

**Authors:** Badsha Sekh, Durgesh Kumar, Hasibur Rahaman, Ravi Shankar Verma, Ramu Maddu, Jianpeng Chan, Wai Lum William Mah, Stuart S. P. Parkin, S. N. Piramanayagam

**Affiliations:** ^1^ School of Physical and Mathematical Sciences Nanyang Technological University Singapore Singapore; ^2^ Indian Institute of Technology Roorkee Roorkee Uttarakhand India; ^3^ Max Planck Institute of Microstructure Physics Halle Germany

**Keywords:** artificial neuron, domain wall, perpendicular magnetic anisotropy, spin‐orbit torque, synthetic antiferromagnet

## Abstract

Neuromorphic Computing (NC) is a promising candidate for Artificial Intelligence (AI) applications. To realize NC, electronic analogues of brain components, such as synapses and neurons, must be designed. In spintronics, domain wall (DW) based magnetic tunnel junctions, which offer both synaptic and neuronal functionalities—are one of the promising candidates. An electronic neuron should exhibit leaky‐integrate‐fire functions, like its biological counterparts. However, most experimental studies focused only on the integrate and fire functions, overlooking the leaky function. Here, we report on a DW neuron device that achieves integration using Spin‐Orbit Torque (SOT)‐induced DW motion and a leaky function via synthetic antiferromagnetic coupling. By fabricating Hall bar devices in a special geometry, we could accomplish these two functionalities. During the leaky process, the maximum DW velocity exceeded 2500 µm/s. Additionally, we investigated the applicability of our neuron devices using a four‐layer Leaky‐Integrate‐and‐Fire (LIF) activated spiking neural network (SNN), achieving 92.57 % accuracy on MNIST and 84.62 % on Fashion‐MNIST (F‐MNIST) using the PyTorch framework. These results further validate the hardware compatibility of spintronic neurons and highlight their strong potential for enabling next‐generation intelligent devices and energy‐efficient neuromorphic computing. The proposed design utilizes materials used in SOT‐MRAM fabrication and is compatible with CMOS fabrication. Therefore, this neuron can be readily integrated into neuromorphic computing.

## Graphical abstract

A spintronic leaky‐integrate‐and‐fire neuron is realized using Spin Orbit Torque driven domain‐wall motion for integration and synthetic antiferromagnetic coupling for the leaky process. The specialized Hall‐bar geometry enables controlled DW dynamics, achieving repeatable integration and firing events. This compact, CMOS‐compatible design highlights a promising route toward energy‐efficient neuromorphic hardware.

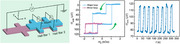

## 1 Introduction

Artificial Intelligence (AI) is widely implemented across various platforms, ranging from social media to astronomy [1, 2, 3, 4]. However, the major problem associated with the present form of AI is its high‐power consumption. As a result, researchers have sought inspiration from the human brain, the most intelligent device with ultra‐low power consumption. For instance, Nvidia 3090 – a high‐performance GPU—consumes ∼ 650 W of power to perform compute‐intensive tasks. In contrast, the human brain only takes 20 W of power to perform similar tasks [5]. Neuromorphic computing (NC) (or brain‐inspired computing) is therefore a promising candidate for AI applications. In the human brain, neurons serve as processors and synapses as memory elements, and they communicate within a neural network. Similarly, electronic analogues of synapses and neurons must be designed to realize NC. Moreover, new algorithms to control the synthetic neurons [6] and synapses must be developed.

In the recent past, several candidates such as phase change memory [7, 8, 9, 10], resistive memory [11, 12, 13, 14, 15], ferroelectric memory [16, 17, 18, 19], and spintronic‐based devices [20, 21, 22, 23, 24, 25, 26, 27, 28] have been investigated for emulating the functionalities of synapses and neurons. Using Among these technologies, spintronics offers the virtues of low‐power consumption, phenomenal miniaturization, and better endurance [29]. Currently, research on quantum materials [30] for spintronic applications provides a new avenue for magnetic storage elements, although challenges remain due to distinct fabrication techniques, low Curie temperatures, and other material limitations. Motivated by these limitations, the search for alternative device platforms has intensified. Within a set of spintronics technologies, spin‐torque/spin Hall nano oscillators have been used as neurons and synapses [31, 32, 33, 34]. Alternatively, domain wall (DW) based magnetic tunnel junctions (MTJ) are also investigated for both synaptic and neuron functions [35, 36, 37, 38, 39]. People have also reported SOT magnetic textures acting as a synaptic element in neuromorphic hardware platforms [40]. Much focus has been dedicated to the development of synapses, while the development of neurons has been overlooked. A SOT‐based neuron device has been proposed through simulations, although the reset process still requires an extra current input to reverse the ferromagnetic magnetization [41]. Moreover, an electronic neuron should demonstrate both the leaky‐integrate‐fire and self‐reset functions exhibited by its biological counterpart. However, most experimental studies lack the demonstration of a leaky (and self‐resetting) function. It has been very difficult to achieve the leaky function experimentally in a manner that can be adapted by the industry. For example, one proposal involves the use of an anisotropy gradient along the length of the DW device to achieve the leaky function [42, 43]. However, an anisotropy gradient can be achieved only using a thickness wedge or a composition gradient created using ion‐implantation, etc. These are not practical methods for CMOS‐based mass‐fabrication technologies. Besides this, People have also explored sigmoidal neuron function due to modulated interfacial Dzyaloshinskii–Moriya interaction via SOT layer selective etching and out‐of‐plane assisted magnetic field, but reset was performed by applying opposite direction pulsed‐field [44]. Another proposal involves the use of shape anisotropy to achieve a leaky function [45]. However, the driving force for leaky function in such devices is not significantly larger than the pinning fields in these materials. As a result, there have not been many experimental demonstrations.

We have been investigating synthetic antiferromagnetic (SAF) coupling‐based [46, 47, 48] neurons for achieving the leaky (and self‐reset) function. Although we achieved the leaky function using a stack of(Co/Pd)_n_/Ru/(Co/Pd)_n_ multilayers, it was difficult to move the DWs in those devices using spin‐orbit torque (SOT) [49]. Quite recently, experimental demonstration of leaky function has been demonstrated via synthetic antiferromagnetic coupling [50], but in this case, the integration function was achieved through Joule heating. There are several inherent limitations in this study through Heating‐driven modulation of the RKKY interaction, which requires significantly higher energy for additional heating element and that can become problematic for dense neuromorphic arrays. Repeated heating cycles can raise reliability concerns due to interfacial diffusion and electromigration, rendering the method incompatible with practical applications. These challenges highlight that realizing SOT‐driven integration together with an intrinsic self‐reset function is still a major bottleneck for practical artificial neuron implementations.

In this report, we describe our work on a Co/Pt‐based SAF neuron device that integrates through spin‐orbit torque and achieves leaky and self‐reset functions via synthetic antiferromagnetic coupling. Moreover, we demonstrated the applicability of our neuron devices by constructing a fully connected SNN in which LIF neurons acted as the activation units. The network exploits the inherent dynamics of LIF neurons to achieve temporal integration and event‐driven spiking behavior. For implementation and evaluation, we used both PyTorch and the SpikingJelly framework, applying supervised training on the MNIST and F‐MNIST datasets. To address the non‐differentiability of spike generation, we employed a surrogate gradient backpropagation method, enabling efficient learning in the spiking domain. The scalability of our neuron device is primarily governed by its excellent Perpendicular Magnetic Anisotropy (PMA) [51]. While large‐scale fabrication may still face practical challenges—such as achieving wafer‐level thickness uniformity, precisely tuning SAF coupling via Ru spacer thickness, and maintaining low edge roughness during high‐volume patterning—these issues are well known in MRAM manufacturing [52]. Importantly, they can be effectively addressed through careful process control of existing industrial routes. Furthermore, the optimized thin film stack deposited on Si/SiO_2_ wafer and the methods for leaky function show a great potential for its compatibility with the CMOS fabrication techniques employed in the STT‐MRAM devices and can be readily employed in NC.

## 2 Micromagnetic Simulations

**FIGURE 1:**
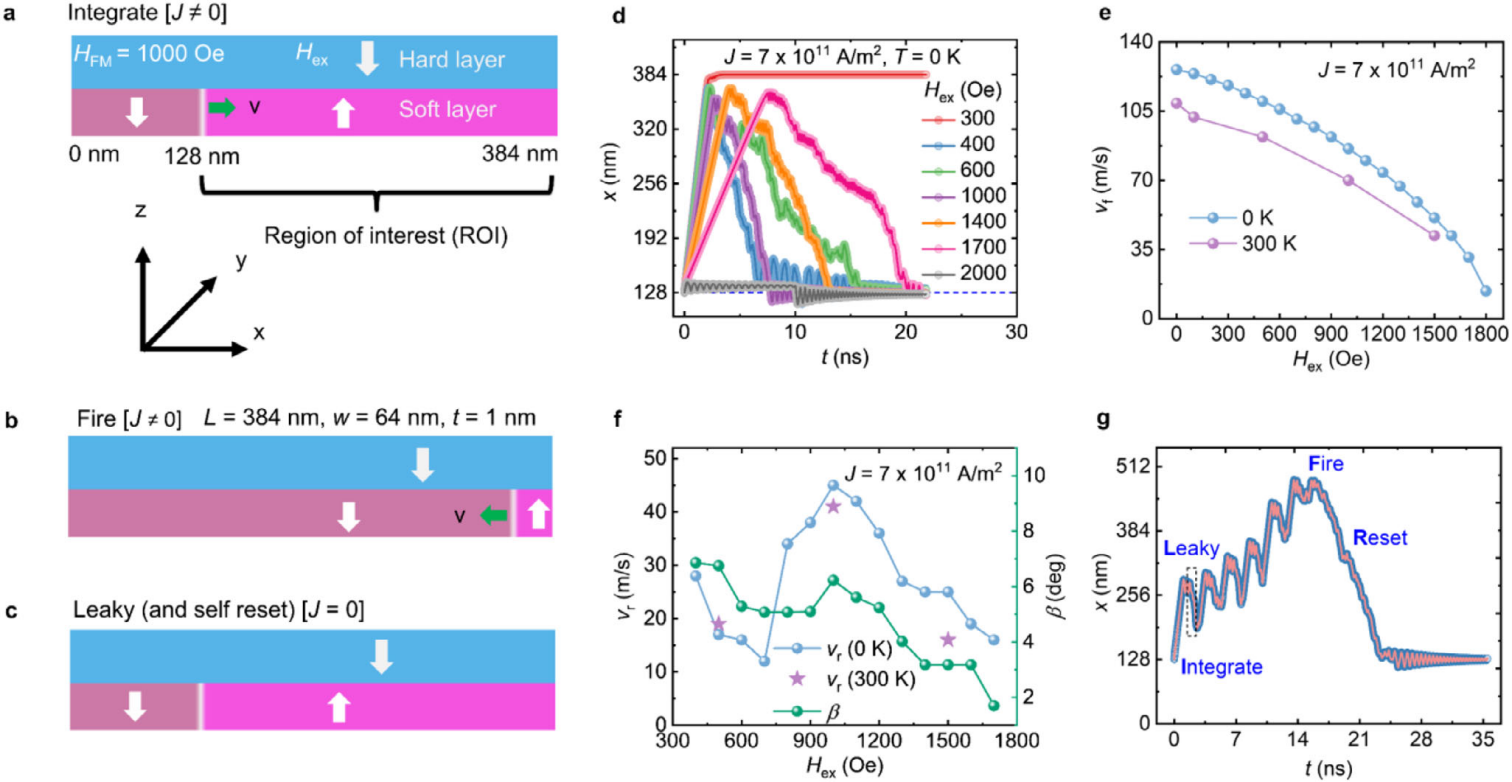
Micromagnetic simulations on neuron devices with synthetic antiferromagnetic coupling: (a–c) The schematic of the studied neuron device. Here, the soft magnetic layer, which carries the DW within the ROI, is synthetic antiferromagnetically coupled with the hard magnetic layer at the top. Initially, the DW can be nucleated at the start of ROI, following the energy minimization of the device. A non‐zero current can drive the DW toward the right end of the ROI. This process is referred to as the integration process. As soon as the DW reaches the right end of the ROI, firing can be achieved. Here, we consider the read probe in the vicinity of the right end of the ROI. Once firing is realized, we switch off the current, and the DW is expected to come back to the initial position following the SAF. (d) The DW position (x) vs. time (t) graph for various values of H_ex_. Here, we have only presented the representative data. (e) The plot of forward velocity (v_f_) as a function of H_ex_ for simulations at 0 K as well as 300 K. (f) The plot of return velocity (v_r_) and angle of DW surface at the start of the reset process for various studied H_ex_. (g) Illustration of leaky, integrate, fire, and self‐reset functions in our studied DW devices.

To systematically understand our proposal of neuron functions through SAF coupling, we first performed detailed micromagnetic simulations. The simulated device is schematically presented in Figure 1a–c. In the proposed DW devices, we moved the DW in the soft magnetic layer. However, the magnetization state remains unchanged in the hard magnetic layer. This layer is used to facilitate the effective exchange field (from the SAF coupling) for the leaky and self‐reset processes. Therefore, we modelled the neuron devices with SAF coupling in the following manner.

We considered a DW device with the dimensions of 384 nm × 64 nm × 1 nm (Figure 1a–c). Here, a part of the DW device (with a length of 256 nm) was considered as the region of interest (ROI) having the SAF coupling. To emulate SAF coupling, we applied an out‐of‐plane (OOP) magnetic field of various magnitudes along the +z direction. This means that we considered a hard magnetic layer on top of the ROI with magnetization pointing along the ‐z‐axis. The remaining 128 nm was considered to have ferromagnetic coupling with the hard magnetic layer. Therefore, we applied a constant OOP magnetic field of 1000 Oe along the ‐z‐direction in this region. The other magnetic and geometric parameters used during the simulations are provided in the methods section.

We utilized spin‐orbit torque (SOT) to achieve the ‘integrate’ function. We studied the DW motion as a function of various relevant parameters such as SAF coupling strength (*H*_ex_), current density (*J*), spin Hall angle (θ_SH_), and longitudinal magnetic field (*H*_x_). Moreover, we also introduced temperature (*T* = 300 K) in our simulations to emulate near‐experimental situations.

To understand the role of *H*_ex_ on the reset (and leaky) process, we studied the forward and return DW dynamics at various *H*_ex_ ranging from 0 to 2000 Oe in steps of 100 Oe. Here, we used *J* = 7 × 10^11^ A/m^2^, θ_SH_ = 0.5, and interfacial Dzyaloshinskii–Moriya interaction (*i*DMI) constant = 0.5 mJ/m^2^. The parameters mentioned above were decided based on our detailed micromagnetic simulations, which are discussed in Section S1.1. All other parameters were kept the same as described in Table 2 of the methods section. As can be seen in Figure 1a, the DW can be moved from the left end of the ROI to the right under the influence of the SOT in all the simulations (except *H*_ex_ ≥1800 Oe). Once the DW reached the right end of the ROI, we switched off the current density and observed the DW dynamics. The pulse durations corresponding to integrate (*t*_ON_) and reset (*t*_OFF_) processes were optimized independently for all the studied cases and are presented in Section S1.2. For *H*_ex_ ≤ 300 Oe, the coupling strength is not capable of resetting the DW position (Figure 1d). For 400 Oe ≤ *H*_ex_ ≤ 1700 Oe, the DW resets to its initial position under the influence of SAF coupling. For *H*_ex_ ≥ 1800 Oe, the DW cannot be pushed forward as the SOT cannot overcome the *H*_ex_. Similar results were observed when we performed these simulations at room temperature. These results are presented in Figure S5a. For deeper insight into these observations, we then plotted the forward velocity (*v*_f_) as a function of *H*_ex_ for simulations performed at 0 K as well as 300 K (Figure 1e). In both cases, *v*_f_ decreases with the increase in *H*_ex_. This is because the opposing torque increases as *H*_ex_ increases. In contrast, with the increase in coupling strength, the return velocity (*v*_r_) first decreases and then increases with a maximum velocity at *H*_ex_ of 1000 Oe. With a further increase in *H*_ex_, the return velocity decreases again. A similar trend was observed for the room temperature simulations. These results are presented in Figure 1f. The observed results are due to at least two factors: (i) the angle of the DW surface at the start of the reset process and (ii) OOP magnetic field‐driven DW motion. Therefore, we first estimated the DW surface angle for various studied cases. As can be seen in Figure 1f, the DW surface angle takes almost a similar relation with *H*_ex_ as the return velocity. A small discrepancy in the trend is attributed to the OOP magnetic field‐driven DW motion (Figure S5b,c).

**TABLE 1:** The parameters utilized in the implementation of the SNN architecture.

Parameters	Threshold Voltage	Reset Voltage	Integration Time Constant	Leak Time constant
Values	214 µV	160 µV	3 ms	3 ms

Finally, we performed micromagnetic simulations to study the leaky, integrate, fire, and self‐reset functions in our devices. For these simulations, we increased the length of the ROI to 384 nm to witness more ‘integrate and leaky’ events. Here, we fixed pulse durations during the integration (*t*_ON‐INT_), leak (*t*_OFF‐LEAK_), and reset (*t*_OFF‐RESET_) at 1, 1.5, and 20 ns, respectively. As can be seen in Figure 1g, we successfully achieved all the above‐mentioned functions in our devices. Moreover, a total of 6 integration and leaky events were recorded on our devices.

## 3 Device Fabrication and Sample Characteristics

**FIGURE 2:**
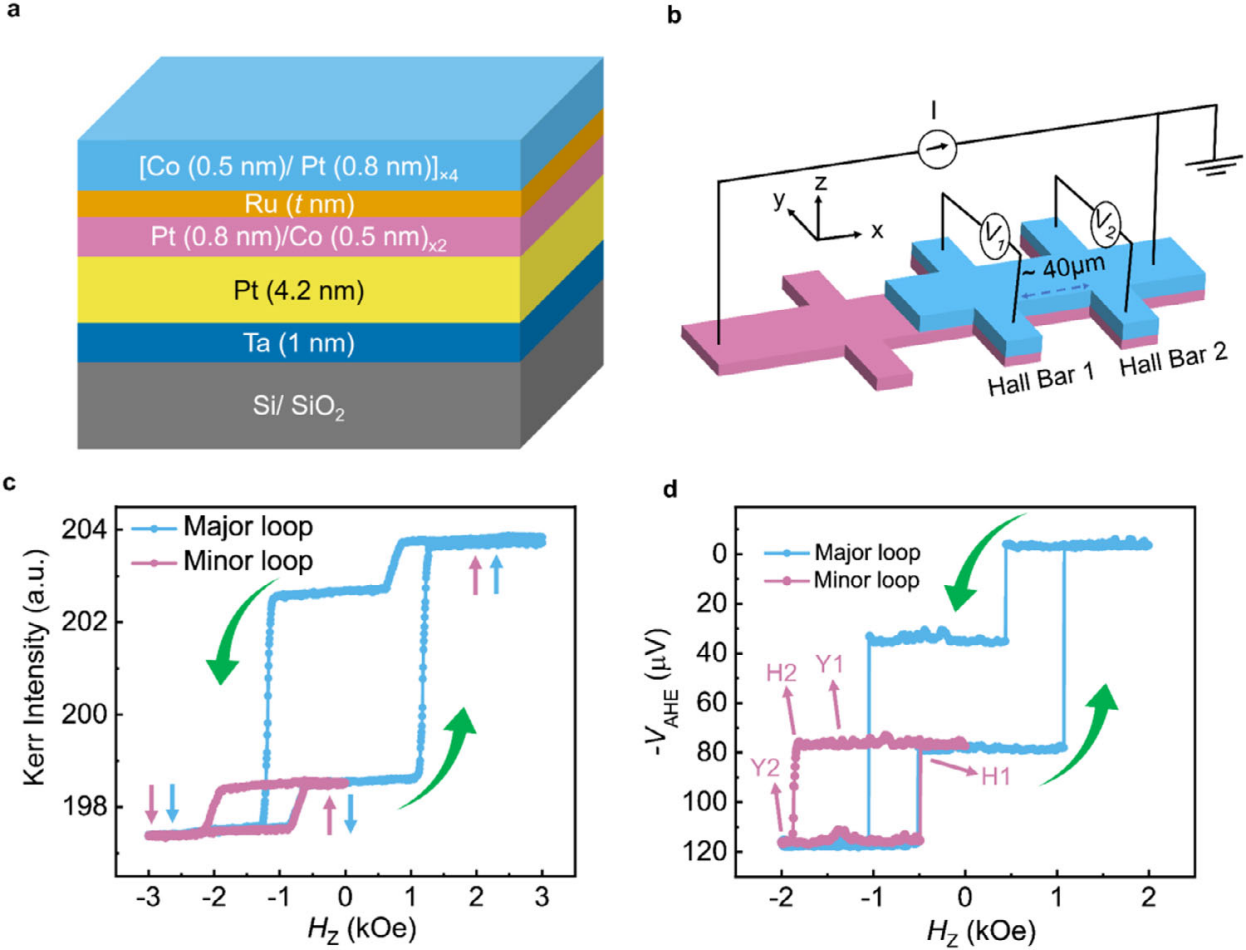
Schematics of the stack, device structure, and its magnetic properties. (a) Stack structure used in this study. Ru thickness was varied from 0.5 to 2.4 nm. (b) The device used for AHE measurement had two regions: the FM layer only and the SAF region, as marked by two different colors. (c) Kerr hysteresis loop of the full stack with a H_ex_ value of 1150 around (for Ru thickness of 2 nm). (d) The AHE measurement of the Hall bar device, where Y1 and Y1 are the AHE voltages at the ground magnetization state and saturation state, respectively. H1 and H2 indicate the switching fields toward the ground magnetization state and saturation state, respectively.

To study the neuron functions [53] experimentally, we deposited synthetic antiferromagnetic (SAF) stacks of the type: Ta (1 nm)/ Pt (4.2 nm)/ [Pt (0.8 nm)/ Co (0.5 nm)]_×2_/ Ru (*t* nm)/ [(Co (0.5 nm)/ Pt (0.8 nm)]_×4_ (Figure 2a) on thermally oxidized SiO_2_ substrates using DC magnetron‐sputtering. A Pt (5 nm) layer, grown on a thin seed layer of Ta (1 nm), was used as the spin‐Hall layer. The free (or soft magnetic) and hard magnetic layers of the SAF stack comprise 2 and 4 repetitions of Co/Pt multilayers, respectively. We varied the Ru layer thickness to achieve the optimal value of the exchange coupling field, *H*_ex_ (Figure S7). The *H*_ex_ vs. *t*_Ru_ exhibited three peaks. For thin values of Ru thickness (0.5 nm), the *H*_ex_ values were very large (>10 kOe). However, for thicker values of Ru (2.55 nm), the *H*_ex_ values were about 1040 Oe.

We characterized our thin film samples using vibrating sample magnetometry (VSM) and polar Kerr microscopy. The VSM results are presented in Figure S8. Figure 2c shows a hysteresis loop obtained by Kerr microscopy for a SAF stack with a Ru thickness of 2 nm. Two kinks were observed in the hysteresis loop due to the exchange field (*H_ex_*) arising from the SAF coupling [54]. The reversal in the first quadrant at around −460 Oe arises due to the switching of the thinner multilayer. At this field, the magnetization of the thicker layer is parallel to the magnetic field, and that of the thinner layer gets aligned antiparallel to the magnetic field. When the magnetic field is further increased, the magnetizations of both layers are oriented parallel to the magnetic field direction due to Zeeman energy [55].

Subsequently, we patterned the thin films into Hall bar devices, as shown schematically in Figure 2b, using optical lithography and Ar ion milling. The Hall bar comprises two regions; region A (left side of the figure) consists only of a soft magnetic layer, whereas region B has the SAF configuration of both the hard and soft layers. The underlying idea behind this design is to insert a DW at the junction of the regions A and B when an applied OOP magnetic field reduces to zero. Once the reversed domain is achieved, the DW can be moved through the SOT generated from the Pt layer. The two Hall bars marked by Hall Bar 1 and Hall Bar 2 at the right end are used to observe the change in magnetization state arising due to the DW motion [56].

Figure 2d shows the anomalous Hall effect (AHE) signal from the patterned Hall bar device that shows a *H*_ex_ value of 1150 Oe. The minor loop denoted by the pink plot corresponds to the switching of the bottom bilayer. The values of *H*_ex_ measured from patterned devices were almost similar to the values measured on the thin films (Figure 2c). Prior to SOT‐driven integration experiments, we performed the integration using an OOP magnetic field. Identical to other cases, the reset was achieved through the SAF coupling. Later, we also performed the integration and reset experiments for multiple cycles and observed good repeatability of the integrate and reset functions in our neuron devices. The corresponding results have been discussed in Section S2.3 in detail.

## 4 Integrate and Leak Functions by Spin‐Orbit Torque and Synthetic Antiferromagnetic Coupling

**FIGURE 3:**
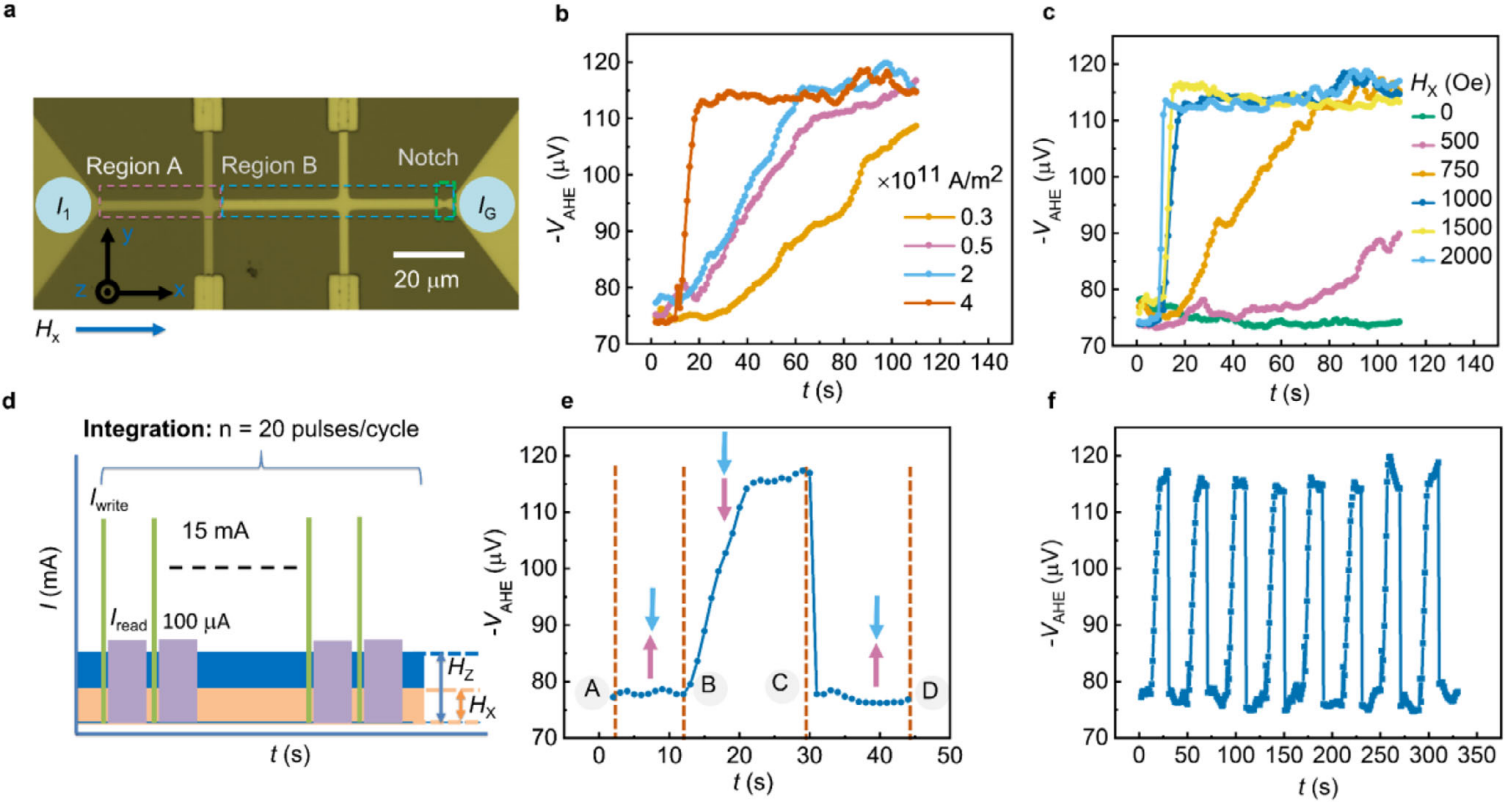
Demonstration of “integrate” and “leak” functions using spin‐orbit torque. (a) Optical image of the patterned Hall bar device. Integrate function (motion of DW and the associated change in the V_AHE_) observed through spin‐orbit torque (b) for various current densities and (c) for various values of H_x_. (d) The schematic of the methodology used for integrate function. (e) The demonstration of integrate and leaky functions of the studied neuron device. (f) SOT‐driven integrate and reset cycles (8 sets) imply the reproducibility of the emulation of neuron activity.

To demonstrate the integration function by spin‐orbit torque [57, 58], we carried out investigations on current‐driven DW motion. Figure 3a shows an optical image of the patterned Hall bar device with the measurement configuration. A notch was inserted at the right end of the device to stop the DW after full integration (device durability with notch insertion has been performed in Section S2.3). Since the exchange field (*H_ex_*) arising from SAF was very high (7100 Oe for a Ru thickness of 1 nm) and SOT cannot overcome this field [59], the integrate function could not be achieved. To overcome this problem, we used a stack of films with a Ru thickness of about 2 nm, wherein the *H_ex_* was found to be lower. Figure 2d shows the AHE loops, indicating that the *H_ex_* is 1150 Oe. From Figure 2d, it can be inferred that an AHE voltage of about −75 µV corresponds to an antiparallel configuration of the soft and hard layers. Whereas an AHE voltage of about ∼ −120 µV indicates a parallel magnetization configuration. Therefore, achieving −120 µV (from a starting point of −75 µV) means full integration in this neuron device. First, we studied the dependence of DW dynamics in the forward direction as a function of different current densities. For this, we applied *H_x_* = 1000 Oe and *H_z_* = −1725 Oe (smaller than the field required to saturate the magnetizations) and current pulses of various magnitudes. In general, one does not need to apply *H_z_* to observe the DW motion. In this device study, the observed *H_ex_* is reasonably high even for 2 nm thick Ru samples, and hence, a certain *H_z_* field was required to overcome the effect of *H_ex_* and to observe noticeable DW motion. For low current density values, DW motion occurs slowly with a gradual change of *V*_AHE_ from −75 to −120 µV. However, the forward DW motion becomes faster as the current density increases. A systematic study of the effect of *H_x_* and *H_z_* revealed that the DW motion does not occur (or slow DW motion) for *H_x_* < 500 Oe (Figure 3c). Moreover, the velocity of DW motion gets higher for higher values of *H_x_* and *H_z_*. The directions of current, *H*_x,_ and DW motion confirm SOT‐induced DW motion. Additionally, we conducted a loop‐shift experiment [60] to observe the SOT properties of our sample stack (Section S2.2). The results of the dependence of DW velocity on assisting OOP magnetic field are shown in Figure S11b.

After demonstrating the integrate function via SOT‐induced DW motion, we proceeded to demonstrate the leak function. Figure 3d shows the schematic of the methodology used. In the presence of an *H_x_* and *H_z_* field, we applied 20 write and read current pulses as shown in Figure 3d. After a certain number of pulses, V_AHE_ increased from −75 to −120 µV. We first recorded the initial state with 10 pulses of only read current. When the write current pulses, *H*_x,_ and *H*_z_ were removed, *V*_AHE_ dropped to about −75 µV, indicating the leak (or self‐reset) function (the corresponding magnetic moment distribution MOKE images at different switching stages have been shown in Section S2.3). This process was repeated for 8 cycles, and in all the cycles, “integrate” and “leaky” functions were observed consistently (Figure 3f). The cycle‐to‐cycle fluctuation ($\frac{\sigma }{\mu }$ ×100) in the firing threshold remains below 1.2 %, and the integration/reset timing is almost the same. Here, σ and µ represent the standard deviation and average value of the AHE voltage (*V*_AHE_), respectively. The nearly identical rising and falling edges confirm that the DW velocity remains consistent over extended operation. Such stability originates from intrinsic material properties—including uniform magnetic anisotropy, stable interfacial characteristics of the Heavy metal/Ferromagnetic stack (such as consistent DMI), and steady SOT efficiency. Additionally, device performance was evaluated at elevated temperatures, as presented in Section S2.3. We also calculated the energy consumption for each cycle of neuron activation to be about 2 mJ and compared it with the power and energy consumption values reported in the literature for different neuron devices (Section S2.4). Meanwhile, reducing the device size and applying ultrafast current pulses [61] are essential steps to further decrease energy consumption for practical neuromorphic computing [62].

Since we have used AHE to sense the magnetization, the *V*_AHE_ increased steadily, and we can call this a step neuron [63]. If MTJ was placed at one end, the measured voltage (or resistance) will change only when the DW reaches the MTJ [64, 65]. In that case, we could observe a spike, and we may call this a spike neuron.

## 5 Return Velocity Investigations in Samples With Various *H*_ex_

Since *H*_ex_ is the driving force for the leaky process [50], it is interesting to estimate the return velocity of the DW in samples with various values of *H*_ex_. For this purpose, samples with different stack structures were prepared by varying the thickness of Ru and Co layers in the bottom bilayers. The details of the samples are presented in Section S2.4. Amongst these, we selected 4 representative samples with *H*_ex_ values of 1500, 1220, 885, and 645 Oe, which were fabricated into Hall bar devices. When we attempted to estimate the return velocity by calculating the time taken for the DW to move to Hall Bar 1 from the notch, the motion was so fast that our setup could not be used for the estimation. Therefore, we applied an opposing OOP *H*_z_ (which is slightly more than *H*_2_) to slow down the return motion. As shown in Figure 2d, *H*_2_ refers to the field below which the magnetization of the soft and hard layers cannot remain parallel. In fact, *H*_2_ should be equal to *H*_ex_‐*H*_c(soft)_, where *H*_ex_ is the exchange field and *H*_c(soft)_ is the coercivity of the soft layer. For various values of *H*_z_, the time taken for the DW to return (from a notch at the end) to Hall Bar 1 was calculated. Since the distance between the notch and Hall Bar 1 was kept at about 88.5 µm, the velocity can be calculated.

**FIGURE 4:**
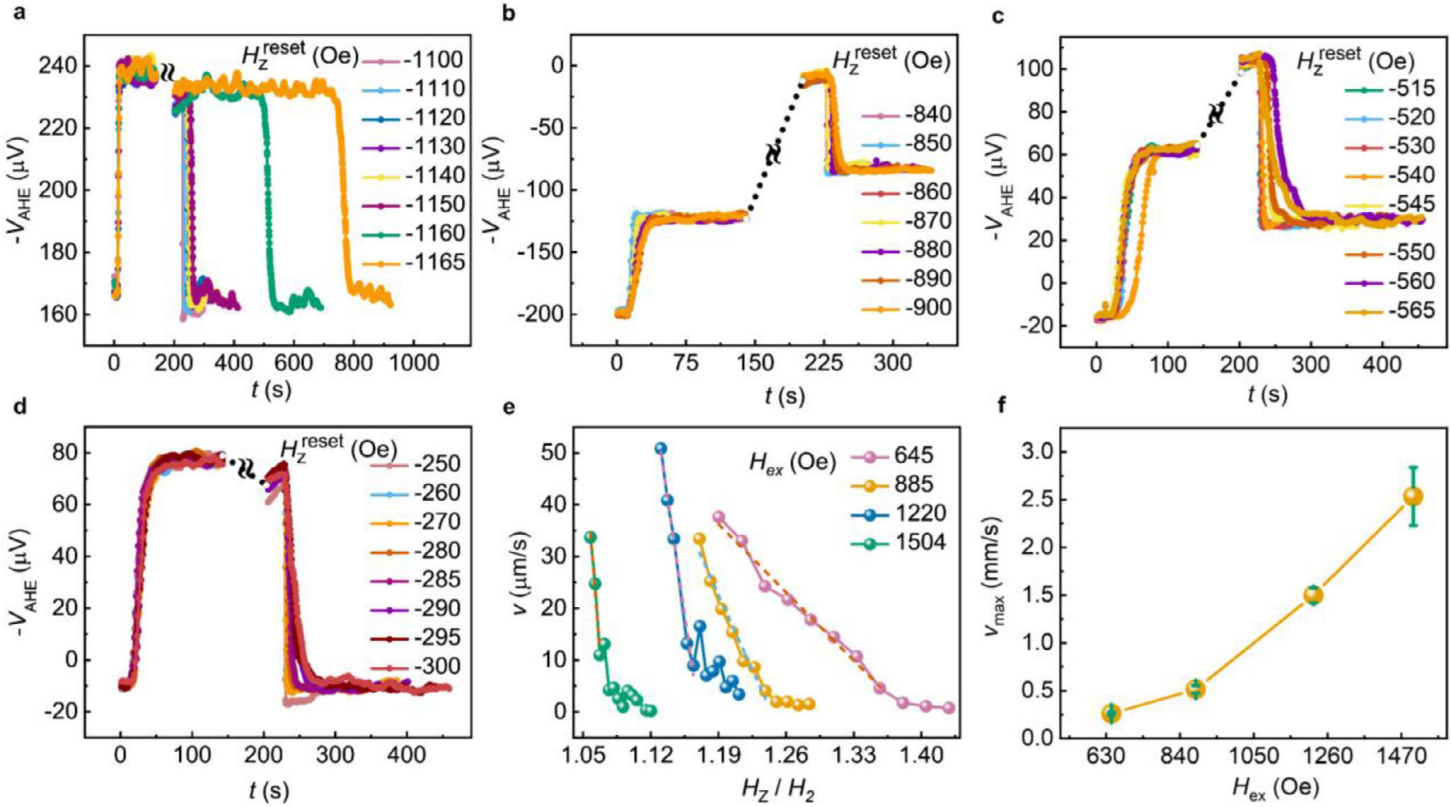
Investigations of the return velocity of the domain walls, which determine the time it takes for the self‐reset process. The AHE voltages used for estimating the return velocity are (a) H_ex_ = 1504 Oe, (c) H_ex_ = 1220 Oe, (d) H_ex_ = 885 Oe, and (e) H_ex_ = 645 Oe. (e) The experimental data (solid lines) and experimental fit (dashed lines) were used for return velocity (v) measurements of samples with various H_ex_. (f) The extrapolated maximum return velocity (v_max_) curve as a function of H_ex_.

As can be seen in Figure 4a, the anomalous Hall voltage (*V*_AHE_) vs. time (*t*) plot provides a glimpse of a typical measurement. For a larger value of *H*_z_ (−1160 Oe), the DW takes a very long time (about 600 s) to move from the notch to Hall Bar 1. This is because the applied *H*_z_ field is larger than *H*_2_ and can stabilize the parallel configuration of magnetic moments in the soft and hard magnetic layers. However, for lower values of the *H*_z_ field (closer to *H*_2_), the DW motion during the reset process becomes faster. We then repeated these measurements on all the above‐mentioned samples, as shown in Figure 4b–d. For a deeper insight into the DW dynamics, we plotted DW return velocity as a function of the applied OOP magnetic field, and the results are presented in Figure 4e. It can be noticed in Figure 4f that the DW return velocity generally shows an increase with *H*_ex_.

## 6 Design and Implementation of a Fully Connected SNN for Image Classification

In this section, we describe our investigations of the effectiveness of the proposed neuron device for NC applications by employing the LIF‐activated SNN. The LIF neuron is a fundamental computational unit in SNNs. The dynamic behavior of LIF is governed by a first‐order differential equation with two key mechanisms: (i) integration of input current, which causes the membrane potential to accumulate, and (ii) leakage, where the potential decays toward its resting level. When the membrane potential exceeds a threshold, the neuron emits a spike, after which the potential is reset to *V_reset_* ​. In the classical LIF model, a single time constant governs the integration and leakage. In contrast, our adaptive (modified) LIF dynamics used in the studied frameworks are expressed as:

(1) $$\begin{array}{c} \;\frac{dV\left(t\right)}{dt}=-\frac{\left(V\left(t\right)-V_{reset}\right)\;}{\tau_{leak}}+\frac{-V\left(t\right)+RI\left(t\right)}{\tau_{integration}} \end{array}$$

where *V*(*t*) is the membrane potential, V_reset_ is the resting potential, I(t) is the input current, R is the membrane resistance. τ_integration_ and τ_leak_ are the integration and leak time constants.

**FIGURE 5:**
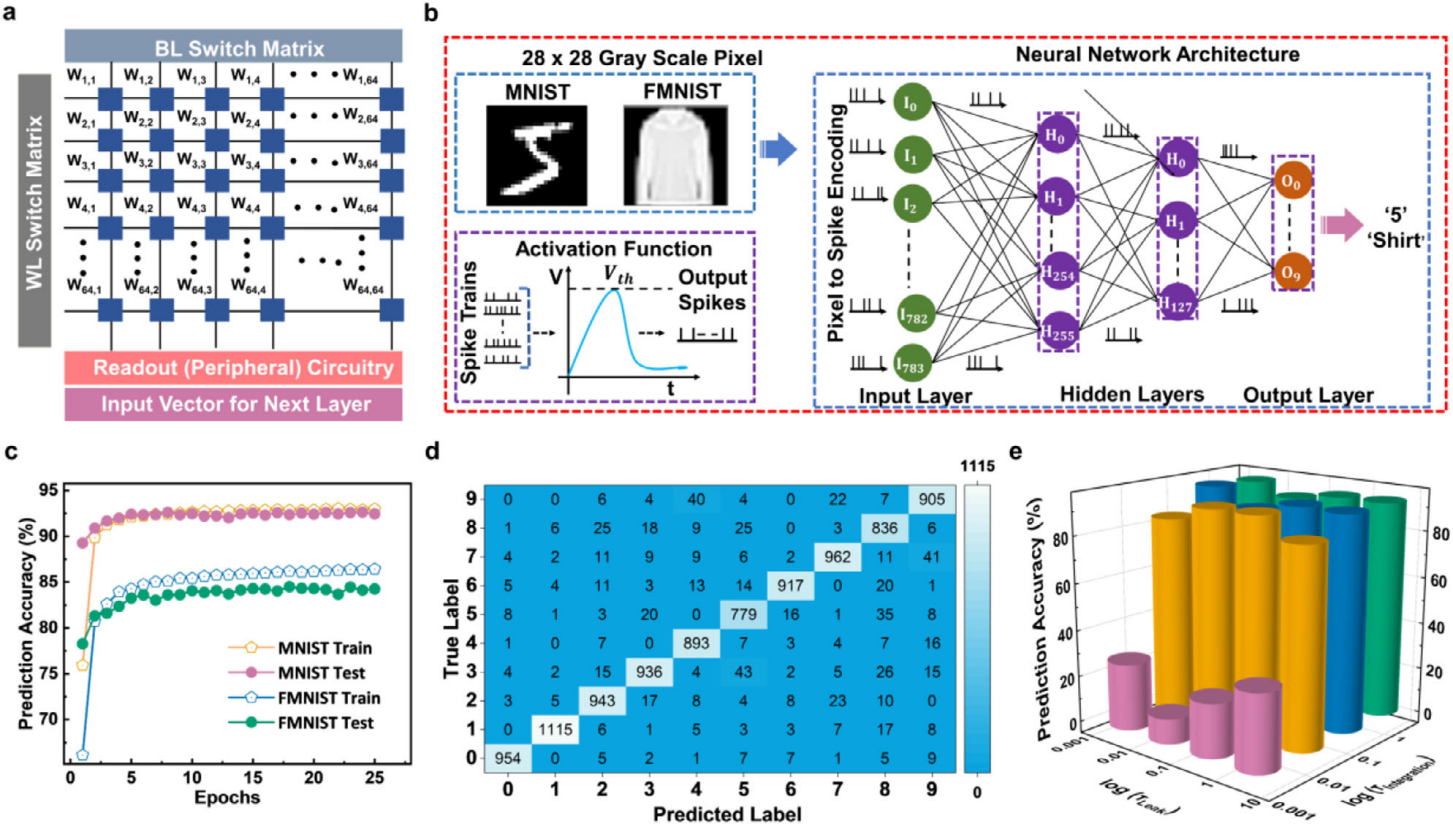
Neural network architecture exploring LIF and SNN for image recognition applications. (a) A schematic representation of the crossbar architecture, which illustrates binary conductance states. The proposed LIF neuronal device integrates the multiply‐accumulate (MAC) computed current along the columns of the crossbar and produces spike outputs according to LIF dynamics. (b) A four‐layer fully connected LIF‐based spiking neural network (FC‐SNN), where the 28 × 28 grayscale images from the MNIST and Fashion‐MNIST datasets are provided as inputs to the first layer. (c) Training and testing accuracy curves for the classification of MNIST and F‐MNIST datasets. (d) Confusion matrices for the MNIST and F‐MNIST datasets, which provide a detailed visualization of classification performance by comparing predicted labels against the True labels of the classes. (e) Performance characterization of neuronal devices using the SNN architecture under varying temporal dynamics, with integration and leak time constants tuned from 3 ms down to 3 µs.

In a discretized form, Equation (1) can be expressed as:

(2) $$\begin{array}{c} V\;\left(t+\Delta t\right)=V\left(t\right)\;\left(1-\;\frac{\Delta t}{\tau_{\mathrm{leak}}}\right)+\frac{\Delta t}{\tau_{\mathrm{integration}}}I\left(t\right) \end{array}$$

where Δ*t* is the simulation time step. Once the membrane potential surpasses the threshold (*V_th_*), neuron emits a spike:

(3) $$\begin{array}{c} S\;\left(t\right)=\left\{\begin{array}{c} 1,\;\;if\;V\left(t\right)\;\geq V_{th}\\ 0,\;\;if\;V\left(t\right)\;<V_{th} \end{array}\right. \end{array}$$

where S(t) is the spike output function of the LIF neuron. The spiking behavior of LIF neurons can be implemented in the neural network architecture using neuromorphic hardware. In such hardware, the crossbar array is a widely used structure for performing parallel multiply–accumulate (MAC) operations directly in memory. It consists of horizontal word lines (rows) and vertical bit lines (columns), with synaptic devices at each crosspoint encoding the connection strength as a conductance *G_ij_*. When an input voltage is applied along the rows, each device multiplies its conductance by the corresponding voltage, generating a weighted current. The total current along each column, following Kirchhoff's law, is given by $I_{j}=\sum_{j}G_{ij}V_{i}$. These synaptic currents serve as inputs to the LIF activation function, which integrates the membrane potential over time and emits spikes when the threshold is exceeded [66, 67]. This event‐driven scheme enables massively parallel and energy‐efficient computation, making crossbar arrays highly suitable for neuromorphic systems, as shown in Figure 5a. To examine the implementation flexibility of neural networks, we first realized the fully connected SNN architecture using an adaptive LIF neuron model in the PyTorch framework and subsequently re‐implemented with the SpikingJelly [68].

Using the above methodology, we implemented digit and fashion classification tasks on an SNN architecture for the standard MNIST and Fashion‐MNIST (F‐MNIST) grayscale datasets. Both MNIST and F‐MNIST consist of 70 000 images (60 000 for training and 10 000 for testing) across 10 classes, with MNIST containing handwritten digit images (0–9) and F‐MNIST representing 10 categories of clothing and accessories. Figure 5b illustrates the neural network architecture designed for image classification, depicting a four‐layer fully connected SNN with 784 input neurons, two hidden layers of 256 and 128 LIF neurons, and 10 output neurons. Each 28 × 28 MNIST/F‐MNIST image is flattened into a 784‐dimensional vector, with pixel intensities encoded into spike trains using Poisson rate coding. The firing probability of each pixel is proportional to its intensity, and repeating this over time steps produces 784 parallel spike trains that serve as input to the SNN.

**TABLE 2:** List of geometric and magnetic properties utilized during the micromagnetic simulations [26].

Parameter	Value
Geometrical parameters	384 nm × 64 nm × 1 nm
Cell size	1 nm × 1 nm × 1 nm
Exchange constant	1.5 × 10^−11^ J/m
Saturation magnetization	1 × 10^6^ A/m
Damping constant	0.012
DMI constant	0.5 mJ/m^2^
Spin Hall angle	0.5
Anisotropy constant	1 × 10^6^ J/m^3^
Easy axis	(0, 0, 1)
FM coupling field	1000 Oe
Temperature	0, 300 K

The training of the SNN for image recognition begins with pixel intensities being encoded into spike trains using Poisson encoding. These spike trains are fed to the input layer and then propagated to the hidden layers of LIF neurons, which integrate incoming currents over time and emit spikes when the membrane potential crosses a threshold, governed by integration (τ_integration_) and leak (τ_leak_) constants [69]. A comprehensive detail of the neuron model and training procedure is presented in Section S2.5. The LIF neuron model incorporates tunable membrane time constants, allowing control over temporal information processing. Fast integration and rapid leakage favor short‐term responsiveness, while slow integration and slow leakage promote longer memory retention. To evaluate how effectively the network adapts and generalizes to novel inputs, we tracked training accuracy over successive epochs and visualized the results. Figure 5c illustrates the training and testing accuracies of the proposed SNN architecture, achieving 94.46 % and 92.57 % on MNIST, and 88.81 % and 83.94 % on F‐MNIST, with both integration and leak time constants fixed at 3 ms as mentioned in Table 1. The network adapts its synaptic weights through spike‐based learning, which is reflected in the training accuracy. The testing accuracy, on the other hand, evaluates how well it generalizes to unseen spike patterns.

Recognition performance is further validated using confusion matrices. Figure 5d shows the matrix for MNIST, where the diagonal entries represent correctly classified spike patterns, and the off‐diagonal entries indicate misclassifications. We have also implemented the confusion matrix for the F‐MNIST, which is provided in Figure S20. The device behavior of the LIF neuron is further explored to check the device's flexibility to work on the variation in the value of the τ_integration_ and τ_leak_ from 3 ms down to 3 µs as shown in Figure 5e. Both datasets demonstrate comparable classification performance, with high training and test accuracies on MNIST and slightly lower accuracy on the more complex F‐MNIST dataset. This confirms the network's capability to generalize across different data domains. The custom PyTorch implementation provides fine‐grained control over neuron dynamics, whereas SpikingJelly offers optimized pre‐built modules that reduce coding overhead and slightly accelerate training. Overall, both frameworks demonstrate efficient event‐driven computation, with SpikingJelly simplifying experimentation while maintaining performance.

## 7 Conclusions

We have reported a DW‐based artificial neuron device that emulates the integration character through SOT‐driven DW motion and successfully exhibits self‐reset functionality via synthetic antiferromagnetic coupling. We have also shown the systematic studies of the DW return velocity variation experimentally through modulating the interlayer exchange coupling. The return velocity increases with respect to *H*_ex_ and reaches the maximum at the highest *H*_ex_ value. In our micromagnetic simulation results, we observed that the forward DW velocity during the integration process decreases with *H*_ex_ due to higher opposing torque at higher *H*_ex_ values. On the other hand, the return velocity variation with *H*_ex_ is related to the angle of DW during the reset process and OOP magnetic field‐driven DW motion. Furthermore, our results provide experimental evidence that the SAF neuron device can be integrated into a neural network for image recognition. The achieved prediction and training accuracies are 94.46 % and 92.57 % on MNIST, and 88.81 % and 83.94 % on F‐MNIST, respectively, demonstrating the potential of SAF‐based hardware platforms for neuromorphic computing applications.

## 8 Methods

### 8.1 Micromagnetic Simulations

To establish a proof‐of‐concept of the “leaky, integrate, fire, and self‐reset” functions in neuron devices with synthetic antiferromagnetic (SAF) coupling, we first performed the micromagnetic simulations using Mumax^3^ software [70]. In the proposed DW devices, we move the DW in the soft magnetic layer. However, the magnetization state remains unchanged in the hard magnetic layer. This layer is used to facilitate the effective exchange field (from the SAF coupling) for the leaky and reset processes. Therefore, we modelled the neuron devices with SAF coupling in the following manner. We considered a DW device with the dimensions of 384 nm × 64 nm × 1 nm. Here, a part of the DW device (with a length of 256 nm; blue region in Figure S1) was considered as the region of interest (ROI) having the SAF coupling. To emulate SAF coupling, we applied an out‐of‐plane (OOP) magnetic field of various magnitudes along the +z direction. This means that we considered a hard magnetic layer on top of ROI with magnetization pointing along the ‐z‐axis. The remaining 128 nm (red region in Figure S1) was considered to have ferromagnetic coupling with the hard magnetic layer. Therefore, we applied a constant OOP magnetic field of 1000 Oe along the ‐z‐direction in this region. The other magnetic and geometric parameters used during the simulations are listed in Table 2.

### 8.2 Thin Film Deposition and Device Fabrication

The SAF film stacks were deposited on thermally oxidized Si (100) substrates via DC/RF magnetron sputtering at room temperature. In the sputtering chamber, the substrate was rotated at 40 rpm during deposition. Prior to the sputtering, the deposition chamber has been pumped to a base pressure of 1 × 10^−8^ Torr. The deposition pressure was maintained at 3 × 10^−3^ Torr during sputtering. After the deposition, multilayers have been patterned into Hall bar devices by optical lithography and Ar ion milling. To nucleate a DW in the soft layer, some portion of the device was etched up to Ru by ion miller. The Ta(5 nm)/Cu(90 nm)/Ta(5 nm) electrodes were deposited using a magnetron sputtering tool for electrical measurements. The device dimensions for field driven and SOT‐driven neuron (two Hall Bar devices as shown in the optical microscopic image in Figure S8) were 20 × 400 µm^2^ and 5 × 150 µm2, respectively. The device dimensions for SOT‐driven return velocity measurements (a three Hall Bar device as shown in Figure 1b) were 5 × 200 µm^2^.

### 8.3 Characterization

The magnetic hysteresis loops of the multilayers were carried out by a Magvision MOKE microscopy. We utilized a Lake Shore 8600 series Vibrating Sample Magnetometer (VSM) for M‐H loops of our samples. The Anomalous Hall effect and magnetization switching measurements were performed using a Keithley 6221 current source and a Keithley 2182 A Nanovoltmeter, which were built into our MOKE system.

### 8.4 Image Recognition

We implemented a spiking neural network (SNN) with Leaky Integrate‐and‐Fire (LIF) neurons to classify the MNIST and F‐MNIST datasets. The network consisted of a 784‐neuron input layer, two hidden fully connected LIF layers (256 and 128 neurons), and a 10‐neuron LIF output layer. Membrane potential dynamics were updated at each discrete timestep, with spikes generated when the threshold was crossed and reset afterward. Training was performed for 25 epochs using backpropagation‐through‐time (BPTT) with a surrogate gradient to approximate the non‐differentiable spike function. Cross‐entropy loss was applied to output spike counts, and parameters were optimized using Adam with a learning rate of 0.001 and weight decay regularization. Accuracy and confusion matrices were recorded per epoch to evaluate performance across both datasets.

## Author Contributions

**Badsha Sekh**: Sample preparation, device fabrication, design of experiments, device testing, analysis, and supported writing. **Durgesh Kumar**: Design of experiments, simulation, device testing, analysis, and supported writing. **Hasibur Rahaman**: Device testing, Simulation, analysis, and was involved in SNN work. **Ravi Shankar Verma**: Design and Implementation of a Fully Connected SNN for Image Classification and supported writing. **Ramu Maddu**: Device testing, analysis, and supported writing. **Jianpeng Chan**: Device testing. **Wai Lum William Mah**: Conceptualization. **Stuart S.P. Parkin**: Device design idea **S.N. Piramanayagam**: Conceptualization of the main idea, design of experiments, analysis, interpretation, and lead writing.

## Conflicts of Interest

The authors declare no conflicts of interest.

## Supporting information




**Supporting File 1**: advs73549‐sup‐0001‐SuppMat.pdf.

**Supporting File 2**: advs73549‐sup‐0002‐VideoS1.docx.

## Data Availability

The data that support the findings of this study are available from the corresponding author upon reasonable request.
