# Supplementary material for: Leaky‐Integrate‐Fire Neuron via Synthetic Antiferromagnetic Coupling and Spin‐Orbit Torque
Source: Adv Sci (Weinh). 2026 Feb 12;13(16):e21732. doi: 10.1002/advs.202521732 (PMC13042506; doi:10.1002/advs.202521732)
Supplement: Supplementary file 1 — Supporting File 1: advs73549‐sup‐0001‐SuppMat.pdf. [file ADVS-13-e21732-s001.pdf]

Supplementary Information

Leaky-Integrate-Fire Neuron via Synthetic Antiferromagnetic Coupling and Spin-Orbit Torque

Badsha Sekh[1], Durgesh Kumar[1], Hasibur Rahaman[1], Ravi Shankar Verma[2], Ramu Maddu[1], Jianpeng Chan[1], Wai Lum William Mah[1], Stuart S.P. Parkin[3] and S.N. Piramanayagam[1,*]

[1]School of Physical and Mathematical Sciences, Nanyang Technological University, 21 Nanyang Link, 637371, Singapore

[2]Indian Institute of Technology Roorkee, Roorkee, Uttarakhand, India – 247667

[3]Max Planck Institute of Microstructure Physics, Weinberg 2, 06120 Halle, Germany

Corresponding author: prem@ntu.edu.sg

Supplementary Information 1: Micromagnetic Simulations

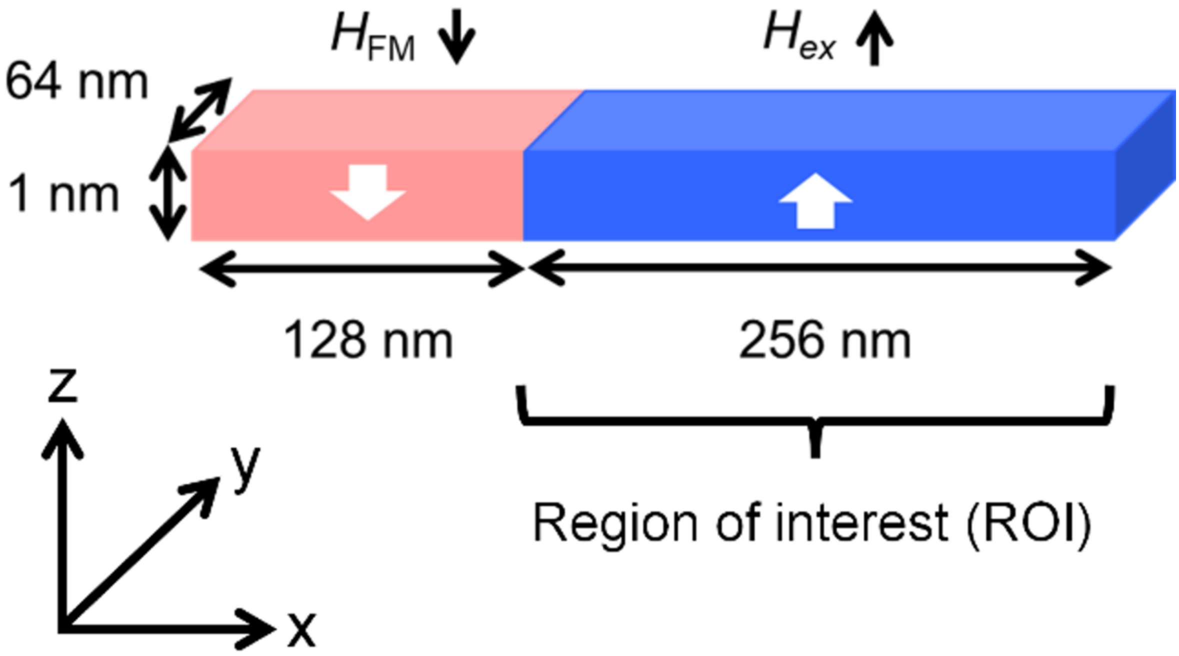

Fig. S1. The schematic illustration of the simulated neuron device with SAF coupling to provide the leaky and reset functions.

In our proposed DW-based neuron device, the DW is driven only within the soft magnetic layer, while the magnetization of the hard magnetic layer remains fixed. The hard layer provides the effective interlayer exchange field required to realize the leaky and self-reset functionalities through SAF coupling.

To model the neuron device incorporating SAF coupling, we divided the simulated DW track into two regions. The first section, a 256-nm-long segment (blue region in Fig. S1), was defined as the region of interest (ROI) where the SAF-mediated exchange field acts on the soft magnetic layer. To emulate this coupling in MuMax3, we applied an OOP magnetic field of varying magnitude along +Z, corresponding to a hard magnetic layer whose magnetization is fixed along -Z.

The remaining 128 nm of the track (red region in Fig. S1) was treated as being ferromagnetically coupled to the hard layer. Accordingly, this region was assigned a constant -Z directed OOP field of 1000 Oe. Together, these two segments reproduce the spatially nonuniform effective field profile expected from an SAF stack. All other geometric and magnetic parameters used in the simulations are summarized in Table 1.

To study the SOT-driven DW dynamics in the proposed devices in detail, we performed micromagnetic simulations using Mumax3, based on the extended Landau Lifshitz Gilbert (LLG) equation[1].

$$\frac{\partial \vec{m}}{\partial t} = -\gamma_0\left(\vec{m} \times \overrightarrow{H_{eff}}\right) + \alpha\left(\vec{m} \times \frac{\partial \vec{m}}{\partial t}\right) + \vec{\tau}_{SOT} \qquad (1)$$

Here, $\vec{m}$ is the locally reduced magnetization, $\overrightarrow{H_{eff}}$ is the effective magnetic field, and α is the Gilbert damping parameter, $\gamma_0 = \mu_0\gamma$, where γ is the gyromagnetic ratio, defined as $\gamma = \frac{ge}{2m_e}$, (g, e, and m_e are Lande factor, electronic charge, and electronic mass, respectively). The effective field can be written as the sum of the total energy of the system, which takes the following form in the present case.

$$H_{eff} = H_{exchange} + H_{anisotropy} + H_{demag} + H_{DMI} \qquad (2)$$

In equation (1), the first term represents effective magnetic field-induced precession of magnetization, and the second term represents magnetic damping, which tends to damp the precession.

The last term, representing the Slonczewski-like torque, where H_{SHE} is the effective SOT field due to SHE[2]. This term is modeled using Slonczewski STT parameters within the Mumax3 simulation framework[3]. The simulation parameters used during the simulations are listed in Table 1, and these are typically observed in experimental research of W/CoFeB/MgO material systems.

Table 1: List of geometric and magnetic properties utilized during the micromagnetic simulations[4].

Parameter	Value
Geometrical parameters	384 nm × 64 nm × 1 nm
Cell size	1 nm × 1 nm × 1 nm
Exchange constant	1.5×10^{-11} J/m
Saturation magnetization	1×10^{6} A/m
Damping constant	0.012
DMI constant	0.5 mJ/m^2
Spin Hall angle	0.5
Anisotropy constant	1×10^{6} J/m^3
Easy axis	(0, 0, 1)
FM coupling field	1000 Oe
Temperature	0, 300 K

In the following section, we describe the role of the key parameters listed in Table 1. In micromagnetic simulations, the geometrical parameters specify the physical size and shape of the magnetic layer. In our simulations, we considered a DW device with lateral dimensions of 384 nm × 64 nm and a magnetic thickness of 1 nm. The simulated device is divided into small rectangular cells (finite-difference grid),

and the magnetization within each cell is assumed to be uniform. Choosing an appropriate cell size is crucial: it must be smaller than the exchange length (l_{ex}) to ensure numerical accuracy of the simulations. In our simulations, the cell size was selected to (1 nm × 1 nm × 1 nm) well below l_{ex} = 4.9 nm.

Precession term

In the LLG framework, the precession of magnetization is governed by the effective field H_{eff}, which is the functional derivative of the total magnetic energy. For the present system, $\vec{H}_{eff}$ can be written as the sum of the fields corresponding to various energies present in the system:

$$H_{eff} = H_{external} + H_{exchange} + H_{anisotropy} + H_{demag} + H_{DMI} \quad (2)$$

The external-field term H_{external} in our simulations includes the interlayer-exchange-induced out-of-plane bias used to mimic the SAF coupling. The exchange field H_{exchange} arises from the isotropic Heisenberg exchange and promotes spatially uniform magnetization. The anisotropy field $H_{\mathrm{anisotropy}}$ is determined by the effective anisotropy

$$K_{\mathrm{eff}} = K_{\mathrm{u}} - \frac{1}{2}\mu_0 M_s^2$$

where K_{u} is the uniaxial (perpendicular) anisotropy constant, and M_s is the saturation magnetization. The effective anisotropy K_{eff} represents the net energy preference for the magnetization to point out-of-plane (OOP) rather than in-plane. It combines the intrinsic interfacial perpendicular magnetic anisotropy (PMA) with the opposing shape (demagnetizing) anisotropy.

The saturation magnetization M_s is the maximum magnetic moment per unit volume that a FM material can achieve when all spins are fully aligned. The demagnetizing field $\mathrm{H_{demag}}$ accounts for dipolar interactions and tends to align the magnetization in the plane. Finally, the Dzyaloshinskii–Moriya interaction contributes the chiral field H_{DMI}, which stabilizes Néel-type DWs with fixed chirality. Together, these terms define the full $\mathrm{H_{eff}}$ governing the precessional magnetization dynamics described by the LLG equation.

It's important to note that the interlayer-exchange field is modelled with an OOP effective field applied as H_{external}. No other external field is applied. The exchange constant A quantifies the strength of the exchange interaction between neighboring spins in a ferromagnet. In addition to isotropic exchange, $\mathrm{MuMax^3}$ incorporates the interfacial Dzyaloshinskii-Moriya interaction (iDMI) as an antisymmetric exchange term. The DMI constant D sets the strength of the chiral interaction at the heavy-metal/ferromagnet interface. A finite D stabilizes a fixed chirality-typically a Néel-type DW. In our simulations, $D = 0.5$ mJ/m^2 is sufficient to stabilize a robust chiral Néel wall.

Damping term

The Gilbert damping constant α characterizes how rapidly the magnetization dissipates energy and relaxes toward its local equilibrium direction. Physically, it reflects the strength of dissipative interactions- such as spin-lattice and spin-electron scattering- that oppose the precessional motion driven by the effective field. In micromagnetic simulations, α strongly influences the dynamics of DW motion, affecting both the speed and stability of the DW during current- or field-driven processes. In our simulations, we employ α = 0.012, a relatively low damping value that is consistent with widely reported experimental measurements for CoFeB/MgO systems. This choice enables realistic modeling of the fast DW dynamics typically observed in ultrathin CoFeB films.

Spin-Orbit term

The third term in the LLG equation represents the torque on local magnetization due to the SOT. In our simulations, we utilized Slonczewski and Berger formalism, which is given as the following, to emulate the SOT. This is a well-established method to apply SOT in the MuMax[3] community.

$$\vec{\tau}_{SOT} = \frac{\beta(\epsilon+\alpha\epsilon')}{1+\alpha^2}\left(\vec{m}\times\vec{m}_p\times\vec{m}\right) - \frac{\beta(\epsilon'-\alpha\epsilon)}{1+\alpha^2}\left(\vec{m}\times\overrightarrow{m_P}\right) \qquad (3)$$

$$\beta = \frac{J_z\hbar}{M_S e d} \qquad (4)$$

$$\epsilon = \frac{P\Lambda^2}{(\Lambda^2+1)+(\Lambda^2-1)(\vec{m}.\overrightarrow{m_P})} \qquad (5)$$

Here, the symbols have the following meaning: J_z: current density along *z*-axis (related to spin current in the context of SOT), *d*: thickness of the magnetic layer, m_P: electron polarization direction (related to the spin polarization in SOT), Λ: Slonczewski parameter, ϵ': secondary spin torque parameter. Here, we have set the secondary spin torque parameter at zero. This means that we are only considering damping like torque in our simulations.

The DW was driven through SOT in all the simulations where *i*DMI is used for deterministic DW motion. Moreover, the DW was inserted at the left end of the device and pushed further using DC/pulse current in all the simulations.

Supplementary Information 1.1

First, we studied the domain wall (DW) dynamics in our neuron devices at different current densities. In these simulations, we fixed the temperature (*T*), synthetic antiferromagnetic (SAF) coupling field (H_{ex}), DMI constant (*D*), and spin Hall angle (θ_{SH}) at 0 K, 1000 Oe, 0.5 mJ/m^2, and 0.5, respectively. The current density (*J*) was varied from 1×10^{11} A/m^2 to 9×10^{11} A/m^2 in the steps of 2×10^{11} A/m^2. At J ≤ 3×10^{11} A/m^2, the spin-orbit torque (SOT) is unable to overcome the opposing torque from the H_{ex}. Therefore, the DW stays at its initial position following an oscillatory DW motion around the initial position. At a current density of 5×10^{11} A/m^2 and above, the DW moves from the left end of the region of interest (ROI) to the right. This signifies the integration function in our neuron devices. As soon as the DW reaches the right end of the devices, we switch off the current density and observe the DW dynamics. During this period, the DW resets back to its initial position under the influence of the synthetic antiferromagnetic coupling. These results are systematically presented in Fig. S2. (a) (and video files 1 and 2). For a clear understanding of the simulation results, we have presented the pulse duration of the integration and reset times at various studied current densities in Fig. S2. (b)

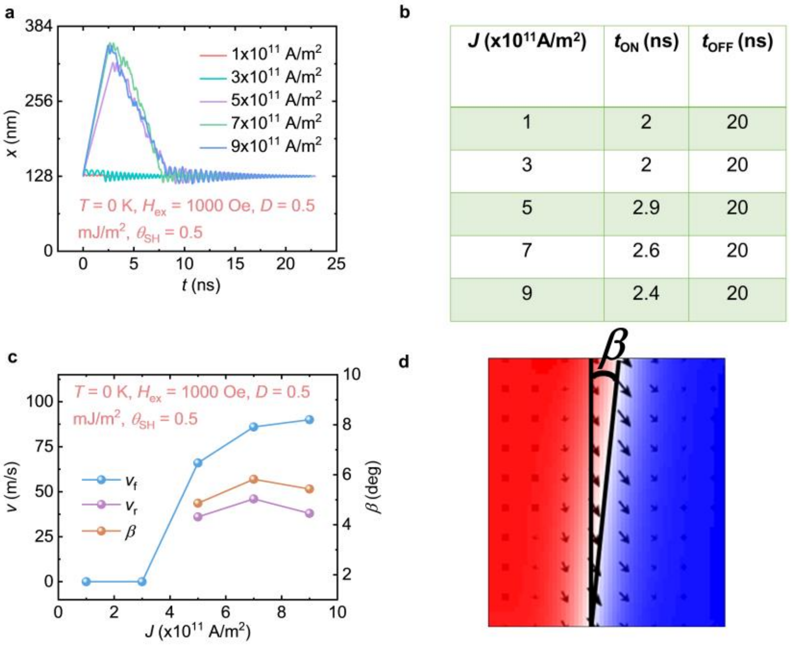

J (x10^{11}A/m^2)	t_{ON} (ns)	t_{OFF} (ns)
1	2	20
3	2	20
5	2.9	20
7	2.6	20
9	2.4	20

Fig. S2. (a) The graph of DW position *vs* simulation time for different current density values. (b) The table, listing the individual pulse durations for integration and reset processes. (c) DW velocity (forward and return) and angle of DW surface as a function of studied current density values. (d) Schematic illustration of DW surface angle at the start of the reset process.

We then plotted the DW velocities during the integration (v_f – forward DW velocity) and reset (v_r – return DW velocity) processes as a function of current density (J) (Fig. S2. (c)). The forward velocity increases as the current density increases, which is an expected result. However, the return velocity first increases and then decreases with maxima at 7×10^{11} A/m^2. To understand these results, we measured the angle of the DW surface at the start of the reset process – defined as β. As we can see in Fig. S2. (c), the DW surface angle takes a similar qualitative trend as for the reverse velocity. We have also schematically shown the definition of DW surface angle in Fig. S2. (d).

Subsequently, we performed micromagnetic simulations at different spin Hall angle values ranging from 0.1 to 0.5 in the step of 0.2. These simulations were performed at a fixed current density of 7×10^{11} A/m^2. The other parameters are identical to the simulations for different current densities. At $\theta_{SH} = 0.1$, the DW stays at its initial position. At the θ_{SH} of 0.3 and 0.5, we can integrate the DW with the help of SOT. Similar to previous simulations, we switch off the current density as soon as DW reaches the right end of ROI and as can be seen in Fig. S3. (a), we successfully achieved the integration and reset processes in above-mentioned cases. These results are also highlighted in video files 3 and 4. The corresponding pulse duration in integration and reset processes are shown in Fig. S3. (b). We further plotted forward and return velocities as a function of θ_{SH} (Fig. S3. (c)). The forward velocity increases as the spin Hall angle increases. Similarly, the return velocity also increases as a function of θ_{SH}. This is again related to the DW surface angle.

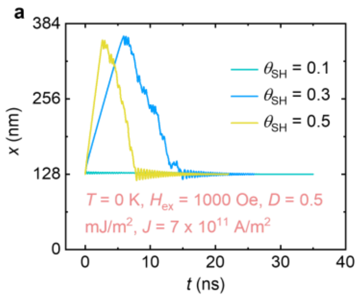

b

θ_{SH}	t_{ON} (ns)	t_{OFF} (ns)
0.1	15	20
0.3	5.9	20
0.5	2.6	20

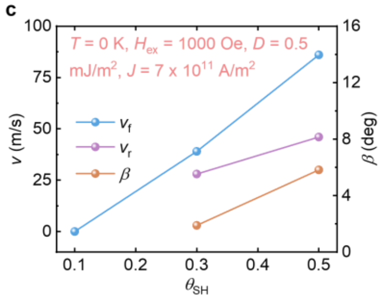

Fig. S3. (a) The graph of DW position *vs* simulation time for different spin Hall angle values. (b) The table, listing the individual pulse durations for integration and reset processes. (c) DW velocity (forward and return) and angle of DW surface as a function of various spin Hall angle values.

To understand the role of longitudinal magnetic field (H_x) on Neuron integration and reset processes, we performed micromagnetic simulations at non-zero longitudinal magnetic field values, and the results are presented in Fig. S4. For all the simulated cases, we can successfully achieve integration and reset functions. Moreover, the forward velocity increases with H_x. Similar to previous cases, the return velocity and DW surface angle are directly proportional for different studied H_x values, with a minimum velocity at 100 Oe of longitudinal magnetic field.

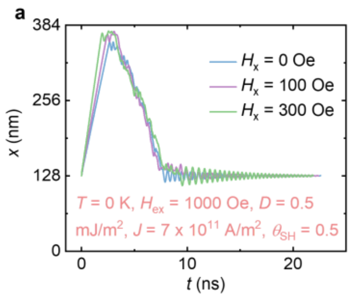

b

H_{ex}	t_{ON} (ns)	t_{OFF} (ns)
0	2.6	20
100	2.4	20
300	1.9	20

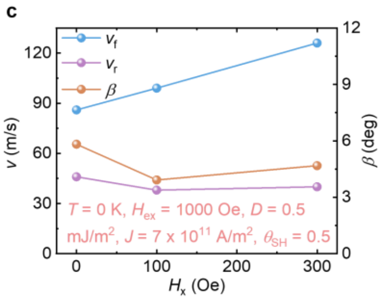

Fig. S4. (a) The graph of DW position *vs* simulation time for different longitudinal magnetic fields. (b) The table, listing the individual pulse durations for integration and reset processes. (c) DW velocity (forward and return) and angle of DW surface as a function of H_x.

Supplementary Information 1.2

Corresponding to the simulation results presented in figure 1 of the paper, here we present relevant complementary results. Related to figure 1(b-c) of the main paper, we first show the DW dynamics for different H_{ex} at room temperature (Fig. S5. (a)). Similar to the simulations at 0 K, a small H_{ex} is not able to bring the DW to the initial state. At higher H_{ex} values, the SOT cannot overcome the torque from synthetic antiferromagnetic coupling. However, for intermediate values of H_{ex}, we successfully simulated the integration and reset functions in our neuron devices.

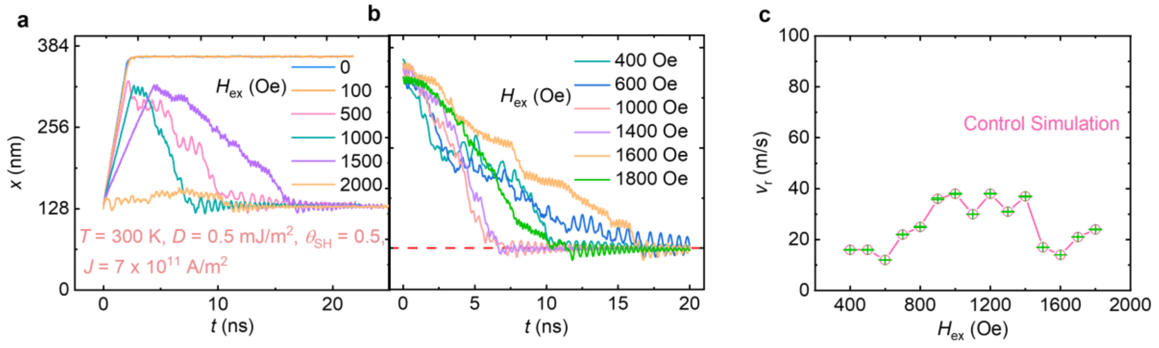

Fig. S5. (a) The graph of DW position *vs* simulation time for different H_{ex} at room temperature. Control simulations: (b) The DW position as a function of simulation time at different H_{ex} values when the DW with β= 0° is introduced at the right end of ROI. (c) The plot of corresponding return velocities at various studied cases.

As mentioned in the paper, there could be at least two contributions to the observed trend in the reverse velocity as a function H_{ex}. (*i*) the DW surface angle at the start of the reset process, and (*ii*) out-of-plane (OOP) magnetic field-driven DW motion. To disentangle these contributions, we inserted a DW at the right end of the devices with β = 0° and observed the DW motion in the influence of H_{ex}. The H_{ex} was varied from 400 Oe to 1800 Oe in the steps of 100 Oe and the results are presented in supplementary Fig. S5. (b). From the DW position *vs* simulation time graphs, we estimated the DW velocity and plotted these as a function of H_{ex} (Fig. S5. (c)). The significance of these results is explained in detail in the paper.

Videos (video files 5-7) for the DW motion at different H_{ex}:

Table S1: A table presenting the time duration for the current pulses during the integration and resent processes.

Temperature (K)	**H_{ex} (Oe)**	**t_{ON} (ns)**	**t_{OFF} (ns)**
0	0	1.8	20
0	100	1.8	20
0	200	2	20
0	300	2	20
0	400	2	20
0	500	2	20
0	600	2.2	20
0	700	2.3	20
0	800	2.5	20
0	900	2.5	20
0	1000	2.6	20
0	1100	3	20
0	1200	3.2	20
0	1300	3.6	20

0	1400	4	20
0	1500	4.4	20
0	1600	5.5	20
0	1700	7.5	20
0	1800	15	20
0	1900	9	20
0	2000	10	20
300	0	1.8	20
300	100	1.8	20
300	500	2	20
300	1000	2.6	20
300	1500	4.4	20
300	2000	10	20

Supplementary Information 1.3

In this section, we present complementary results for the demonstration of leaky-integrate-fire and self-reset functions in our neuron devices based on synthetic antiferromagnetic neurons. The corresponding pulse durations are shown in the following figure (supplementary figure 6). Here, we demonstrated neuron functions at T = 0 K as well as 300 K.

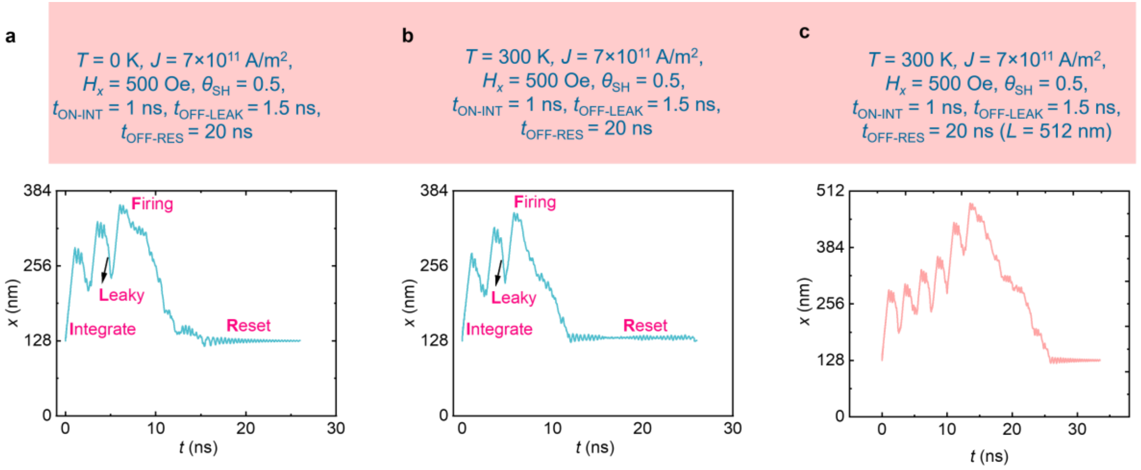

Fig. S6. The demonstration of leaky, integrate, fire, and self-reset functions for (a) the length of ROI (L) = 256, T = 0 K, (b) the length of ROI (L) = 256, T = 300 K, and (c) the length of ROI (L) = 384, T = 0 K.

Videos (video files 8-9) demonstrating the leaky-integrate-fire and self-reset functions in our neuron devices.

The video files are available here:

Video 1: https://youtu.be/RTI6_pwRka0

Video 2: https://youtu.be/YI6gOFCPU-g

Video 3: https://youtu.be/vAAkL7XSmb8

Video 4: https://youtu.be/AbKoAYouGeE

Video 5: https://youtu.be/TeWCq_ne0Uo

Video 6: https://youtu.be/vslKFDgUke0

Video 7: https://youtu.be/0kQMIRMXH5g

Video 8: https://youtu.be/ALP4kqH_dp0

Video 9: https://youtu.be/6zX2lWGsqIg

Supplementary Information 2: Experimental section

Supplementary Information 2.1

In order to study the Ruderman–Kittel–Kasuya–Yosida (RKKY) oscillation[5–7], the Ru interlayer spacer thickness has been varied from 0.35 to 2.55 nm by keeping other multi layers thicknesses unchanged. To check the magnetic properties of these deposited samples we used Mag Vision Kerr microscopy system. As for Ru interlayer spacing 0.45 nm and 0.5 nm, the magnetization state is not saturated at the highest OOP applied field limit of our MOKE set up, we used Lake Shore 8600 series Vibrating Sample Magnetometer (VSM) to find the H_{ex}. The first, second and third maxima peak of H_{ex} was found for Ru thickness 0.5, 1 and 2.1 nm respectively as shown in the H_{ex} versus vs Ru thickness plot (Fig. S7. (h)).

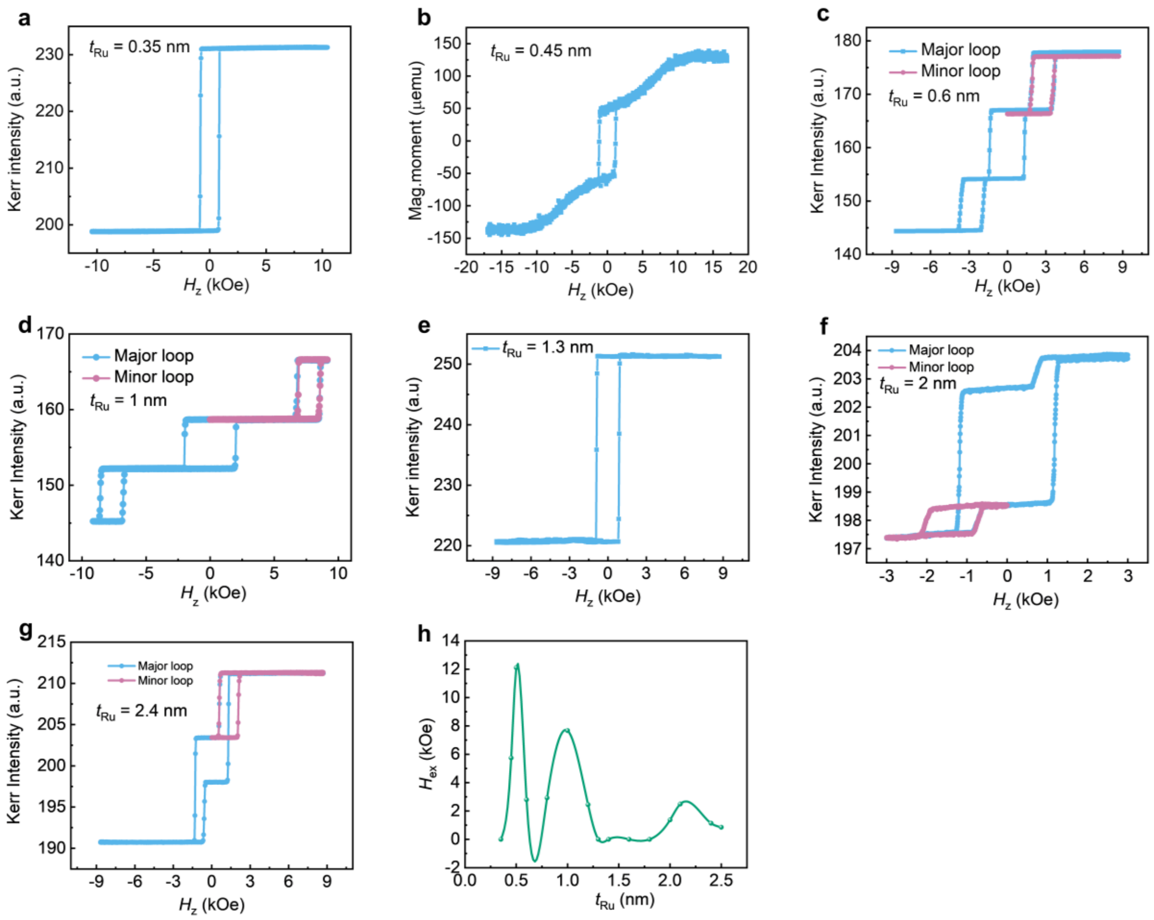

Fig. S7. (a) – (g) Perpendicular hysteresis loops showing the interlayer exchange coupling (IEC) field for different Ru thicknesses. (h) The variation of IEC field (H_{ex}) with respect to the various Ru thicknesses (t_{Ru}) has been plotted.

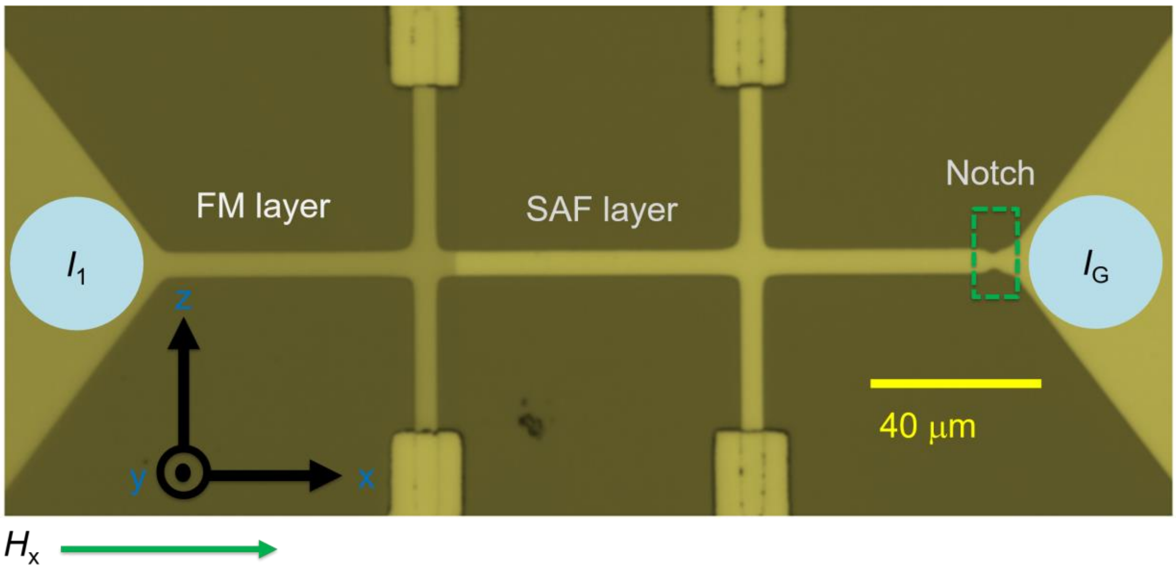

Fig. S8. MOKE optical image of the SAF heterostructure patterned into a Hall bar geometry (20 µm×400 µm), H_x showing the applied in plane field direction. A notch has been inserted at the right end of the SAF layer to stop the domain wall after full integration.

The exchange energy (J_{ex}) for the Ru 1 nm spacer layered SAF stack has been calculated as $J_{ex} = M_s t H_{ex}$ = 0.51 erg/cm^2 , which is comparable to the literature reported value of Co/Ni multilayer SAF stack[8]. Here M_s is the saturation magnetization (720 emu/cc), t is the soft layer thickness (1 nm) and H_{ex} is the interlayer exchange coupling field (7100 Oe).

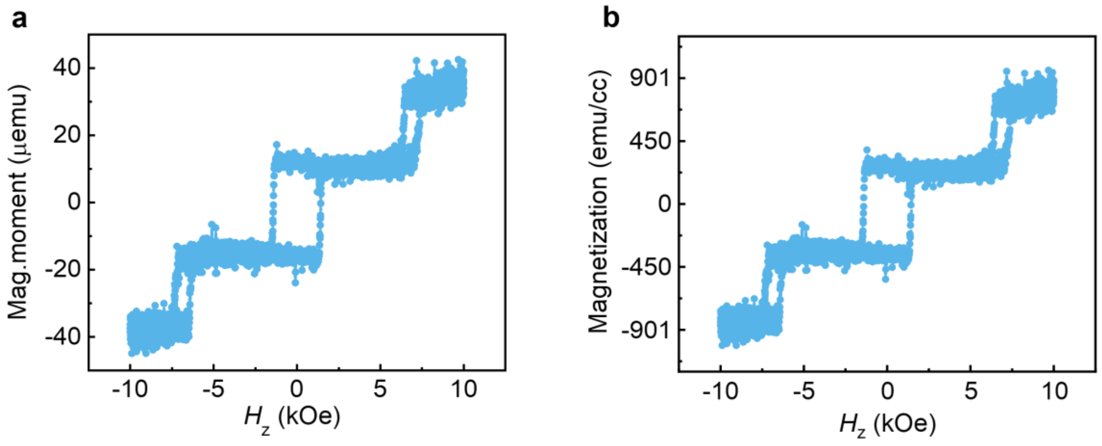

Fig. S9. Out of plane VSM hysteresis loop measurement of Si/Ta(1)/Pt(4.2)/[Co(0.5)/Pt(0.8)]$_2$/Ru/[Co(0.5)/Pt(0.8)]$_4$

In order to determine the magnetization, a Vibrating Sample Magnetometer (VSM) is used at room temperature. Fig.S9. shows the SAF behaviour of the sample under the out of plane applied magnetic field.

Supplementary Information 2.2

To quantify SOT switching efficiency, we performed loop-shift experiment on our free-layer stack Si/SiO_2/Ta (1 nm)/Pt (5 nm)/Co (0.5 nm)/Pt (0.8 nm)/Co(0.5 nm)/Ru(1 nm) using Kerr Microscopy. Under a constant in-plane field (H_x), and constant DC, we recorded the AHE signal by varying the OOP H_z field. The in-plane field acts as a symmetry-breaking field. Due to the Spin-Hall effect (SHE) in Pt heavy metal, an effective out-of-plane (OOP) magnetic field will be generated, which can switch the magnetic layer. Fig. S10. a-c demonstrate the opposite shifting of AHE loop for changing polarity of DC, under H_x = 1600 Oe. The direction of loop-shift supports the SOT switching behavior of Pt as found in the literature[9]. The observed shift (ΔH) increases with increasing H_x (Fig. S10. d).

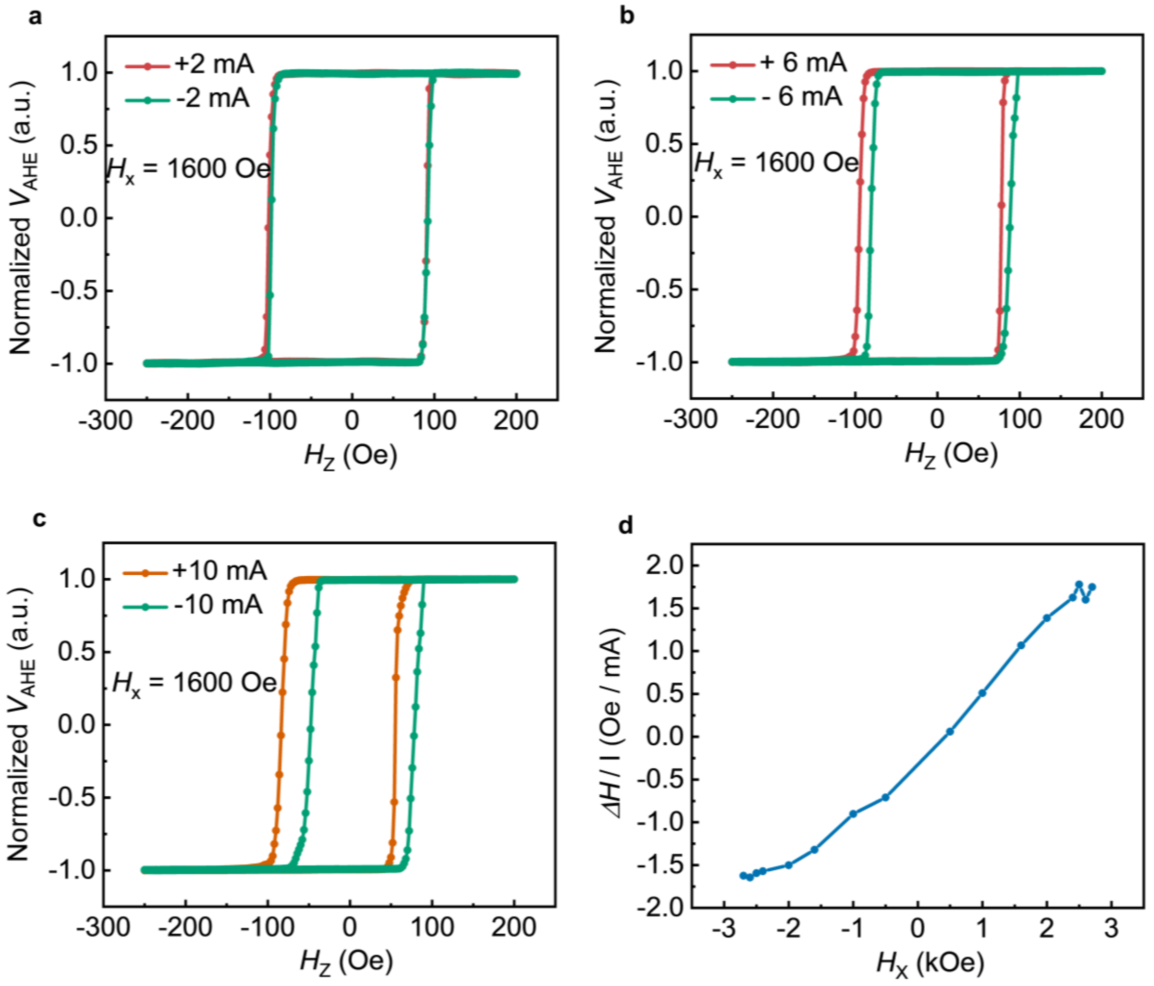

Fig. S10. (a)-(c) The AHE loops with H_X = 1600 Oe and applied current of ±2 mA, ±6 mA and ± 10 mA on the Si/SiO_2/Ta(1 nm)/Pt(5 nm)/Co(0.5 nm)/Pt(0.8 nm)/Co(0.5 nm)/Ru(1 nm) sample, respectively. (d) The shift of the AHE loops (ΔH) due to SOT under different H_X.

Supplementary Information 2.3

Field driven Neuron characteristics:

The integration behaviour of the hall bar device was performed by the external applied out of plane (OOP) magnetic field and the self-reset functionality achieved through RKKY exchange field. Initially, the domain wall nucleation formed by applying a very high negative OOP magnetic field and then turned off the field to zero. Then we varied the OOP field near the switching field of the thinner layer in such a way that it integrates and reaches to the saturated magnetization state. The saturated AHE voltage was found to be around -1250 µV which corresponds to the parallel magnetization state of both the soft and hard layers. For these measurements, the AHE voltage was observed by applying the

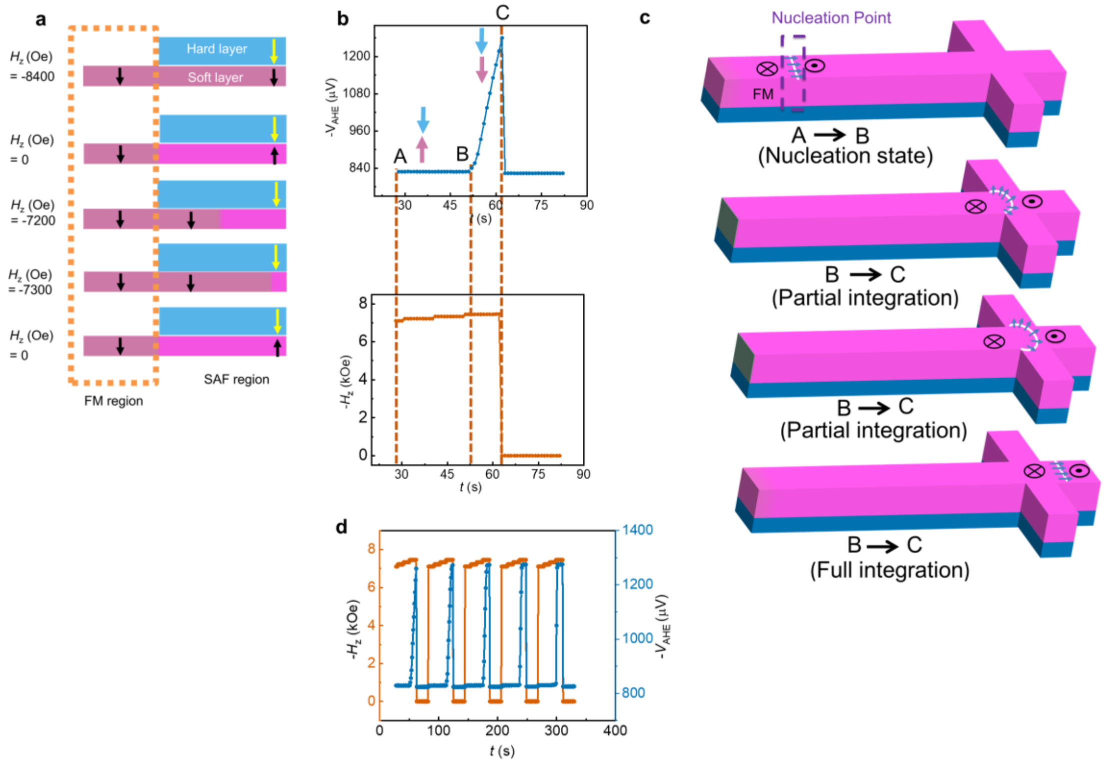

Fig. S11. (a) Schematic diagrams of the domain wall motion under different OOP magnetic field. (b) The integration and self-reset function exhibition by sweeping the field in the AHE measurement. (c) The domain wall nucleation and integration indicated conceptually in the SAF hall bar Neuron device. (d) The five repetition cycles emulating the Neuron characteristics.

read and write current pulses of 1 mA with 200 ms pulse duration. The domain wall returns (reset) to the nucleation region after the OOP field reaches to zero. The AHE voltage at this rest state was found to be around -830 µV which corresponds to antiparallel magnetic moments of the soft and hard layers. The schematic illustration of the domain wall motion during this process has been shown in Fig.S11. (a). In one full cycle experiment, the domain wall stays at initial state (A to B), the integration happens at B to C and finally reset occurs at C (Fig.S11. (b)). The domain wall position during nucleation, integration and reset process has been shown schematically in a Hall bar design (Fig.S11. (c)). To examine the repeatability of the Neuron characteristics, the five repetition cycles were continued as shown in Fig.S11. (d)).

SOT driven Neuron characteristics:

As discussed in the main paper Figure 3. (a), from the AHE loop of the new SAF device having Ru thickness 2 nm (Fig.S12 (a)) H_{ex} calculated was found to be 1150 Oe. To expand the domain having -z magnetization, we applied the H_x (1000 Oe) and current (4×10^{11} A/m^2) in the same direction varying H_z assistance field from 0 Oe to -2000 Oe (as shown in Fig. S12. (b)). The mimimum H_z assistance field required for integration process was found to be -1725 Oe which is less than the switching field of the

soft layer. We investigated the integration and self-reset function in a single cycle and performed in a successive 8 repetitive cycle where both integration and self-reset was observed (Fig. 3 (f)). We first saturated both the FM and SAF region by applying a very high negative OOP field. Then we applied 10 reading pulses (I_{read} = 100 µA, duration of I_{read} = 100 ms). This is the initial state where domain wall remains unchanged. On the other hand, when 20 pulses of writing current = 15 mA followed by I_{read} = 100 µA with H_x = 1250 Oe, H_z = -1725 Oe applied the domain wall propagates implying the expansion of -z magnetized domain. While the domain wall moves back to its initial state due to H_{ex} by switching off the external driving force assisting the integration. We applied 20 I_{read} pulses to record the magnetization in the reset process.

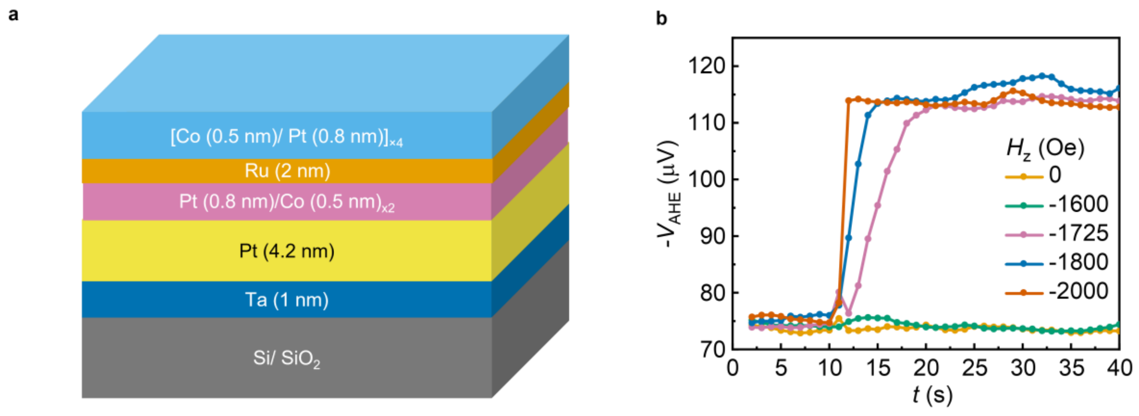

Fig. S12. In the Current induced SOT driven Neuron device measurements experiments. (a) Schematic of SAF heterostructure used. (b) Integration function with various H_z when a constant H_x = 1000 Oe and writing current density 4 ×10^{11} A/m^2 was applied.

Reliability Assessment of the Lithographically Defined Notch: The notch is introduced at the end of the Hall bar (2.5 µm away from one end) solely as a geometric pinning site, ensuring that the domain wall stops at a well-defined position once the integration process is completed. This notch (as shown in Fig.S13. (a)) is patterned using the same lithographic and etching steps as the rest of the device, without introducing any additional processing stages. Notches of this type are routinely employed in domain-wall devices, racetrack memories, and artificial synapses to control DW position[10,11]. Such lithographically defined notches have been widely demonstrated to be robust under repeated current-driven operation[11].

Maximum pinning strength is achieved when the notch is sufficiently deep relative to the wire width, but not so extreme that it distorts the DW structure. Moreover, in the notched region the local cross-section is reduced, which elevates the localized current density and Joule heating. In our neuron device, the notch height is kept around 50% of the wire width. To mitigate heating and current crowding, we intentionally designed the notch with smoother, which preserves geometric pinning while reducing current crowding. Furthermore, we employ well-separated current pulses (1 ms) to

minimize thermal load. At the applied current density of 2 × 10^{11} A/m², we did not observe any degradation in the device characteristics after 35 repetition events as shown in Fig. S13.(b)

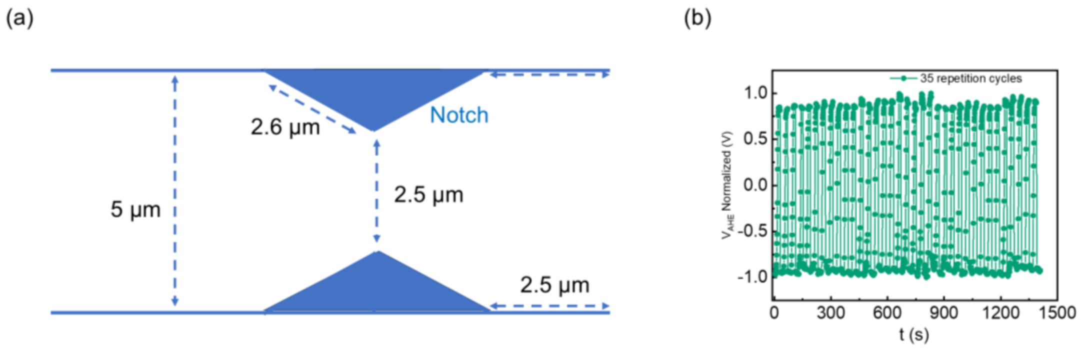

Fig. S13. *(a)The schematic illustration of the notch used in our neuron Hall bar device (5 μm width, 150 μm length). (b) Device performance of 35 repeated neuron-operation cycles.*

Domain wall motion during SOT induced integration and self reset due to H_{ex}: We first saturated the whole device with large out-of-plane magnetic field, then at the remanence state of the device, a magnetic DW is inserted between the junction of soft layer (left side region of device) and full SAF region (right side region of device). After the DW insertion, in the initial state of the device configuration, we captured the Kerr image (Fig. S14. (a)) with keeping the Hall bar region as region of interest.

Here the grey and black colour represents two oppositely oriented magnetic domains of the soft layer. Thereafter, we applied 100 consecutive write current pulses of I = 30 mA along with H_x = 1000 Oe and H_Z assistance field (-1725 Oe) to drive the DW deterministically and integrate the device through SOT. After the application of each current pulse, we recorded the Kerr images, which demonstrates the real-time DW dynamics.

Fig. S14 (b)- (d) shows the DW motion and partial integration process at different instances of time. The integration process completes once the DW passes through the Hall cross region. Fig. S14. (e) depicts the full integration state, where the DW reaches another end (pinned at the notch), the DW device become fully integrated. It's important to note here that since we have applied assisting OOP field to overcome the opposing exchange field during integration, besides of SOT, this OOP field also influences the integration process, as reflected in the switching of the voltage channel in Fig. S14. (d) – (e). After 100 pulses of write current, we removed the external current pulses, H_X, H_Z assistance field and DW returns back to its initial configuration as shown in Fig. S14. (f).

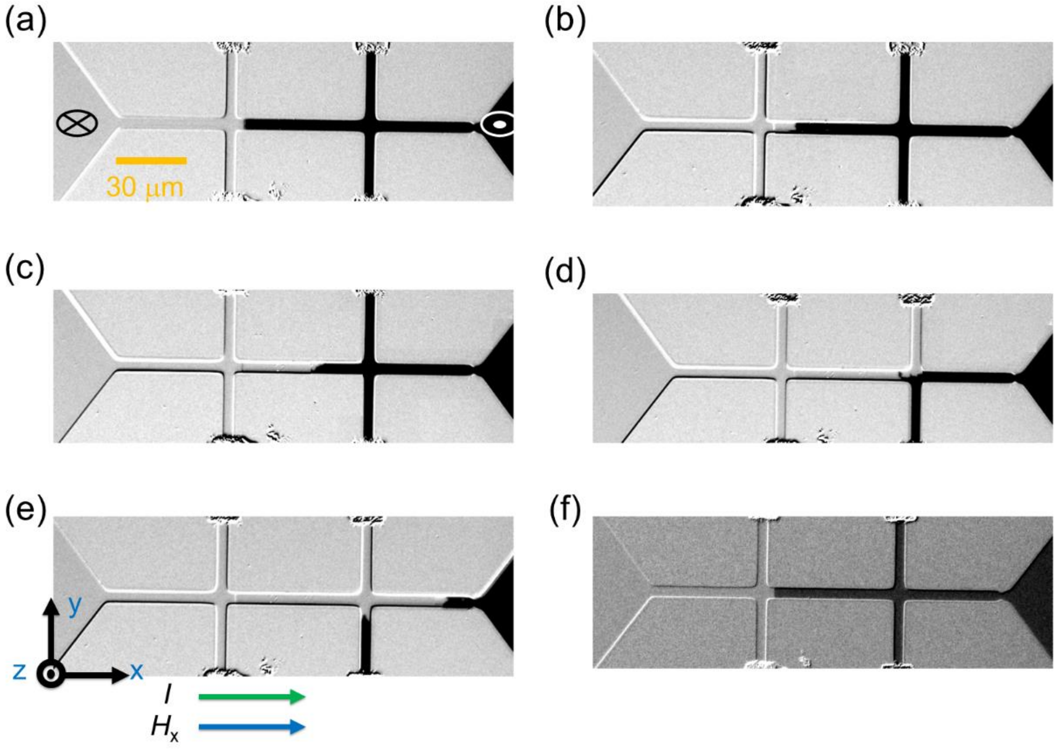

Fig. S14. (a) Kerr image *of* DW insertion between soft layer and SAF region at the remanent magnetization state*.* (b)- (d) Domain wall movement during Partial integration with different applied write current pulses*.* (e*)* Kerr image at full integration of the neuron device. (d) The Kerr image at reset when the DW moves back to the initial state.

Device performance under varying temperature conditions : Here we demonstrate the integration and reset performance of our SOT neuron device (H_{ex} = 1150 Oe) under different operating thermal conditions (Fig. S15 (a)- (g)). The temperature was varied from room temperature (RT) to 55 °C. During integration, a 30 mA write current with 100 pulses were applied, while H_Z and H_X were kept the same values as used in Fig. 3(b). To analysis the temperature dependent behaviour, we measured $\frac{\Delta V\ (T)}{\Delta V\ (T_0)}$ across all the performed thermal conditions. Here $\Delta V\ (T)$ indicates the changes of the V_{AHE} at different temperature (T°C) and $\Delta V\ (T_0)$ denotes the changes of V_{AHE} at RT. The device resistance does not change much in temperature range up to 55°C. The SOT-driven integration functionality was maintained up to 45 °C, yielding a similar AHE voltage change as observed at RT as shown in Fig. S15. (h). Although the integration process becomes slower while increasing the temperature. This behaviour is likely due to reduced SOT efficiency at elevated device temperatures[12], leading to slower domain-wall motion and thus a smaller change in V_{AHE}. Nevertheless, the reset performance remained consistent across all temperatures up to 55 °C, indicating the robustness of the built-in H_{ex}, which reliably drives the domain wall back to its initial nucleation site. We did not extend the temperature range beyond 55 °C because the integration performance became significantly weaker at this point.

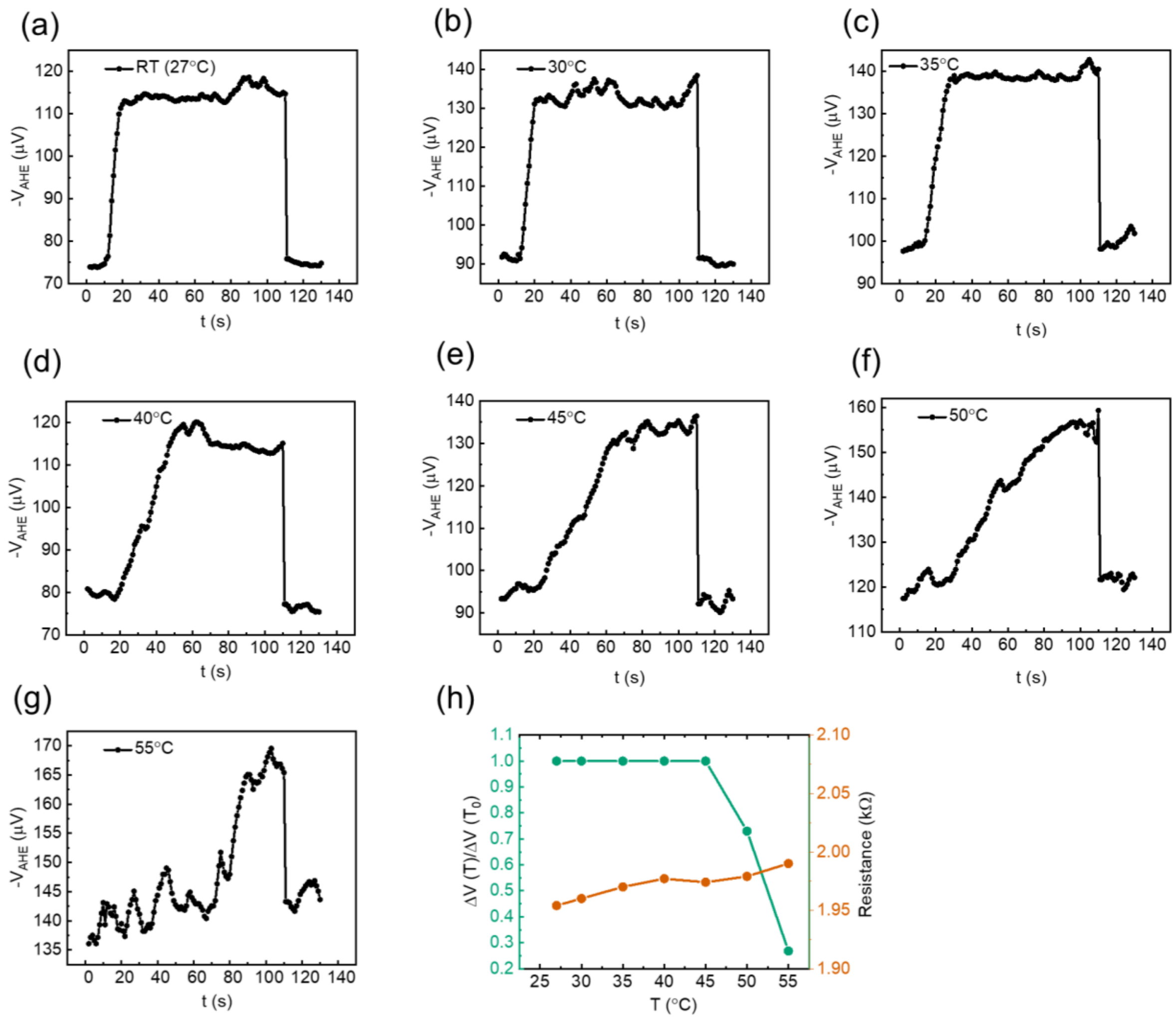

Fig. S15. Device functionality test at various operation temperatures. (a)-(g) integration and self reset function at various temperature (RT, 30°C, 35°C, 40°C, 45°C, 50°C, 55°C). (h) Comparison of change of V_{AHE} values and device resistance at different temperatures.

Supplementary Information 2.4

Table S2: Details of the samples with different stack structures prepared for return velocity experiments.

Sample Name	Sample Stack	H_{ex} (Oe)
S1	Ta(1)/Pt(4.2)/[Pt(0.8)/Co(0.6)]$_{\times2}$/Ru(0.95)/[Co(0.6)/Pt(0.8)]$_{\times4}$	2746 (Film)
S2	Ta(1)/Pt(4.2)/[Pt(0.8)/Co(0.8)]$_{\times2}$/Ru(0.95)/[Co(0.8)/Pt(0.8)]$_{\times4}$	5264 (Film)
S3	Ta(1)/Pt(4.2)/[Pt(0.8)/Co(0.8)]$_{\times2}$/Ru(0.95)/[Co(0.6)/Pt(0.8)]$_{\times4}$	2400 (Film)
S4	Ta(1)/Pt(4.2)/[Pt(0.8)/Co(0.6)]$_{\times2}$/Ru(2.59)/[Co(0.6)/Pt(0.8)]$_{\times4}$	2773 (Film)
S5	Ta(1)/Pt(4.2)/[Pt(0.8)/Co(0.8)]$_{\times2}$/Ru(2.59)/[Co(0.6)/Pt(0.8)]$_{\times4}$	2231 (Film)
S6	Ta(1)/Pt(4.2)/[Pt(0.8)/Co(1)]$_{\times2}$/Ru(2.59)/[Co(0.6)/Pt(0.8)]$_{\times4}$	1600 (Film)
S7	Ta(1)/Pt(4.2)/[Pt(0.8)/Co(1)]$_{\times2}$/Ru(2.5)/[Co(0.6)/Pt(0.8)]$_{\times4}$	1573 (Film)
S8	Ta(1)/Pt(4.2)/[Pt(0.8)/Co(1)]$_{\times2}$/Ru(2.47)/[Co(0.6)/Pt(0.8)]$_{\times4}$	1780 (Film),<br>1504(Device)
S9	Ta(1)/Pt(4.2)/[Pt(0.8)/Co(1)]$_{\times2}$/Ru(2.4)/[Co(0.6)/Pt(0.8)]$_{\times4}$	1600 (Film),<br>1220(Device)
S10	Ta(1)/Pt(4.2)/[Pt(0.8)/Co(1)]$_{\times2}$/Ru(2.35)/[Co(0.6)/Pt(0.8)]$_{\times4}$	1060 (Film),<br>645 (Device)
S11	Ta(1)/Pt(4.2)/[Pt(0.8)/Co(1)]$_{\times2}$/Ru(2.33)/[Co(0.6)/Pt(0.8)]$_{\times4}$	262 (Film), 885 (Device)

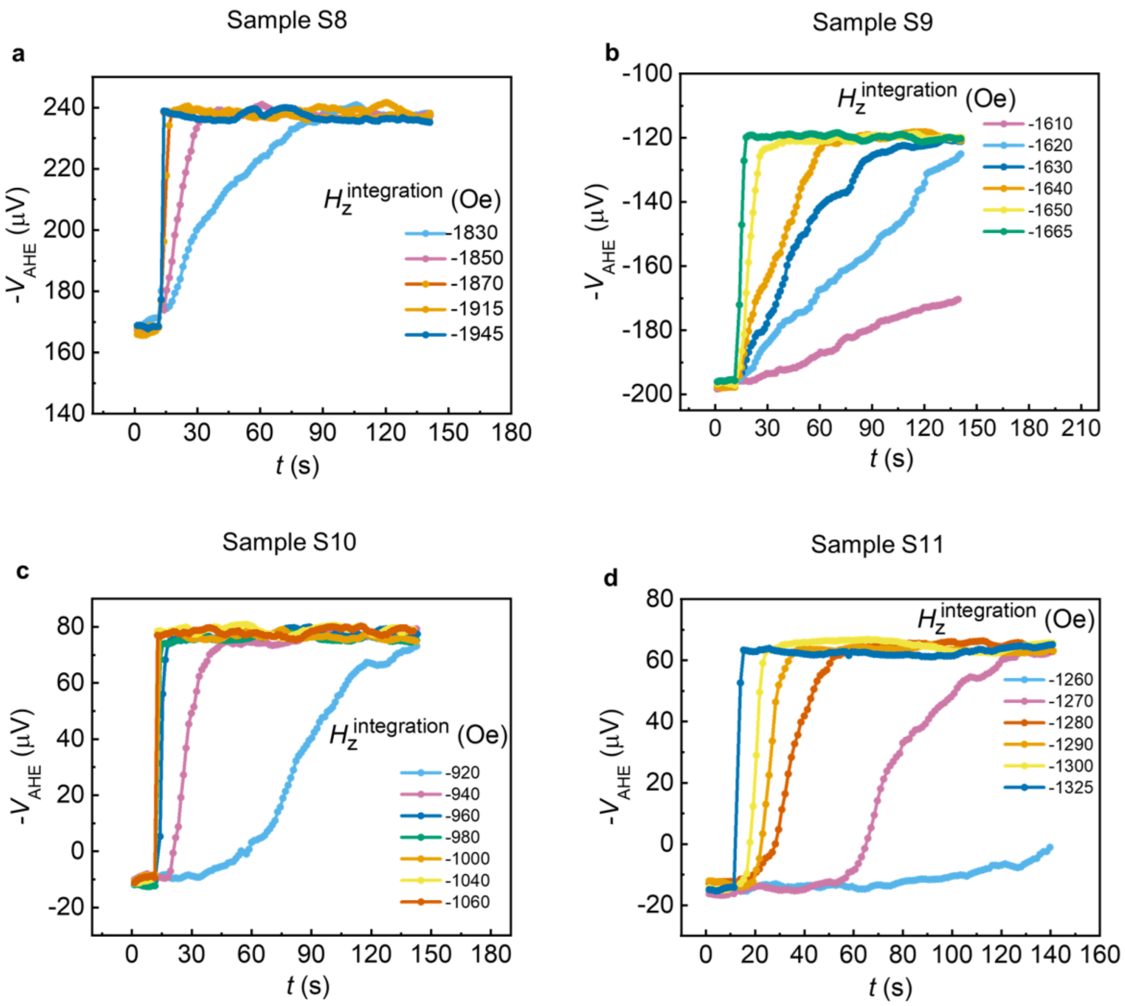

Fig. S16. Integration function for different samples having different H_{ex} with various H_z when a constant H_x = 1000 Oe and 30 mA writing current was applied in all cases. The AHE voltage for integration process with (a) Sample S8 (H_{ex} = 1504 Oe), (b) Sample S9 (H_{ex} = 1220 Oe), (c) Sample S10 (H_{ex} = 645 Oe), (d) Sample S11 (H_{ex} = 885 Oe).

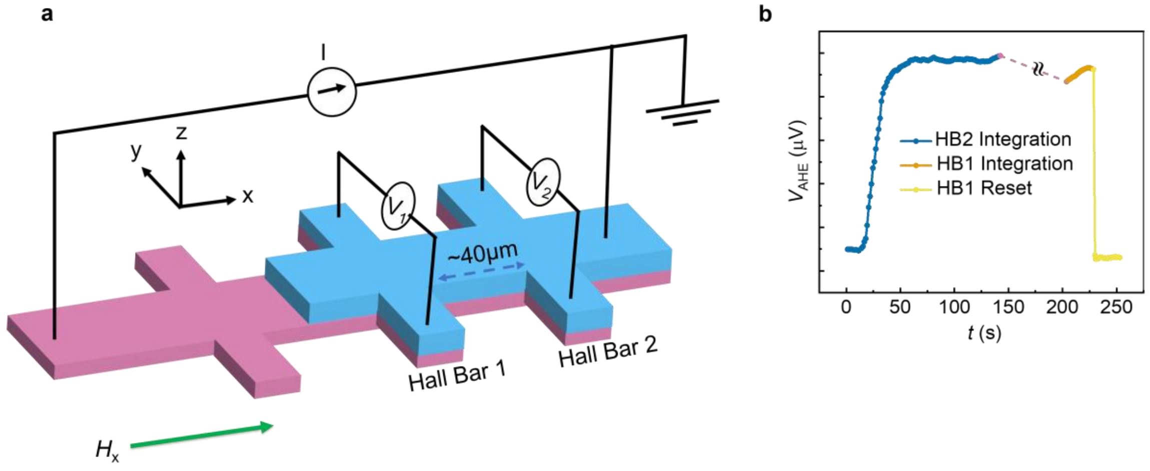

Fig. S17. In the return velocity experiments (a) Hall bar schematic view, where Hall bar 1(HB1) and Hall bar 2 (HB2) are placed on the SAF region, H_x showing the applied in plane field direction. (b) Anomalous Hall Effect (AHE) measurement scheme during Integration at HB2 and HB1 and the changes in AHE signal at HB1 during reset process.

Table S3: The H_z assistance field applied for integration process prior the return velocity measurements.

Sample Name	H_z field for integration (Prior return velocity)
S8	-1850 Oe
S9	-1630 Oe
S10	-940 Oe
S11	-1280 Oe

Sample S8

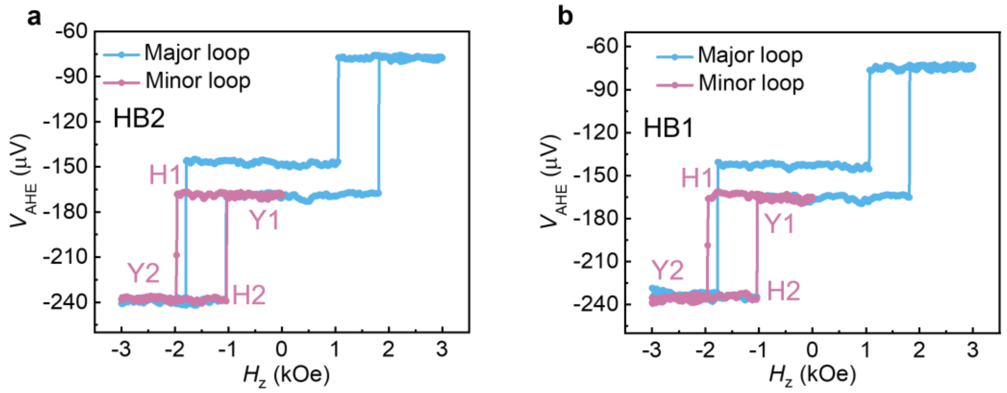

Sample S9

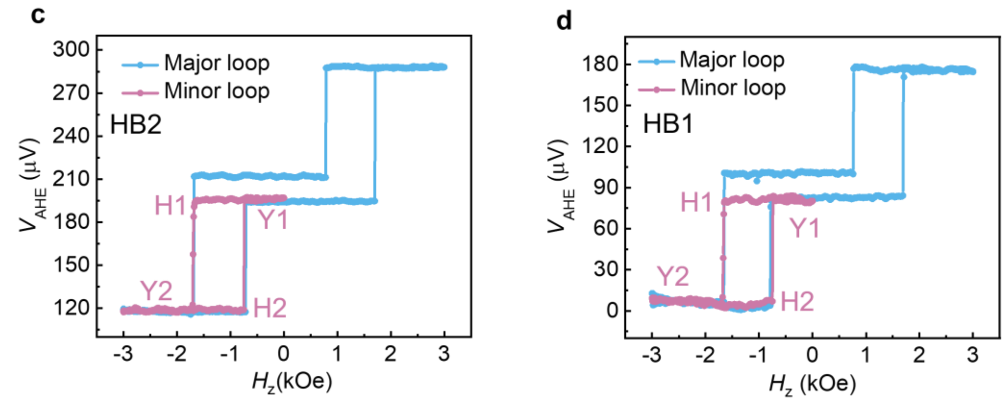

Sample S10

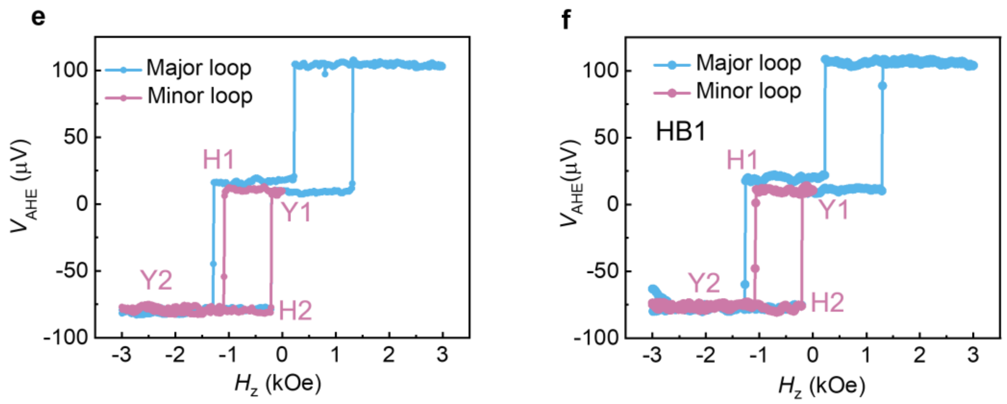

Sample S11

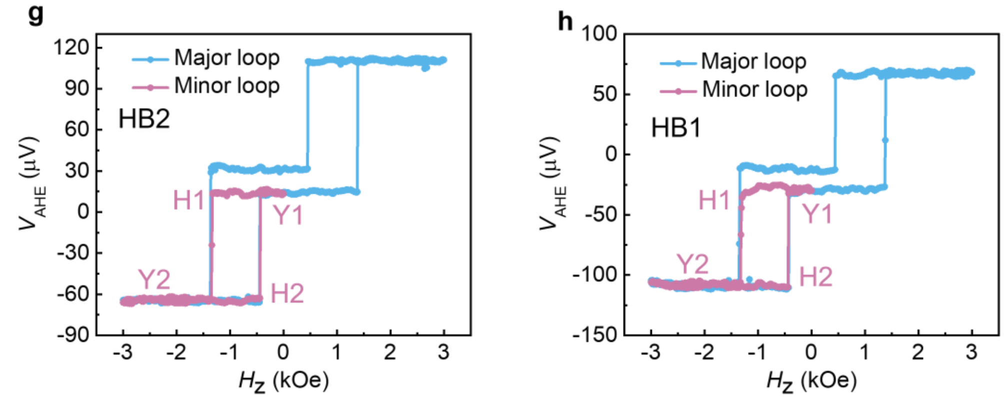

Fig. S18. AHE measurement at HB2 and HB1. Each row is for one sample. (a-b) Sample S8, (c-d) Sample S9, (e-f) Sample S10, (g-h) Sample S11.

Table S4: Device results of AHE measurements at two Hall Bars (HB1 and HB2) for four different samples having various $H_{ex.}$

Sample Name	Hall Bar 2 (HB2)	Hall Bar 1 (HB1)
S8	H1 = -1975 Oe, H2 = -1040 Oe<br>Y1 = -168 μV, Y2 = -238 μV	H1 = -1951 Oe, H2 = -1040 Oe<br>Y1 = -163 μV, Y2 = -235 μV
S9	H1 = -1680 Oe, H2 = -745 Oe<br>Y1 = 195 μV, Y2 = 120 μV	H1 = -1674 Oe, H2 = -743 Oe<br>Y1 = 80 μV, Y2 = 8 μV
S10	H1 = -1080 Oe, H2 = -210 Oe<br>Y1 = 10 μV, Y2 = -80 μV	H1 = -1075 Oe, H2 = -210 Oe<br>Y1 = 10 μV, Y2 = -75 μV
S11	H1 = -1335 Oe, H2 = -434 Oe<br>Y1 = 15 μV, Y2 = -65 μV	H1 = -1300 Oe, H2 = -440 Oe<br>Y1 = -27 μV, Y2 = -110 μV

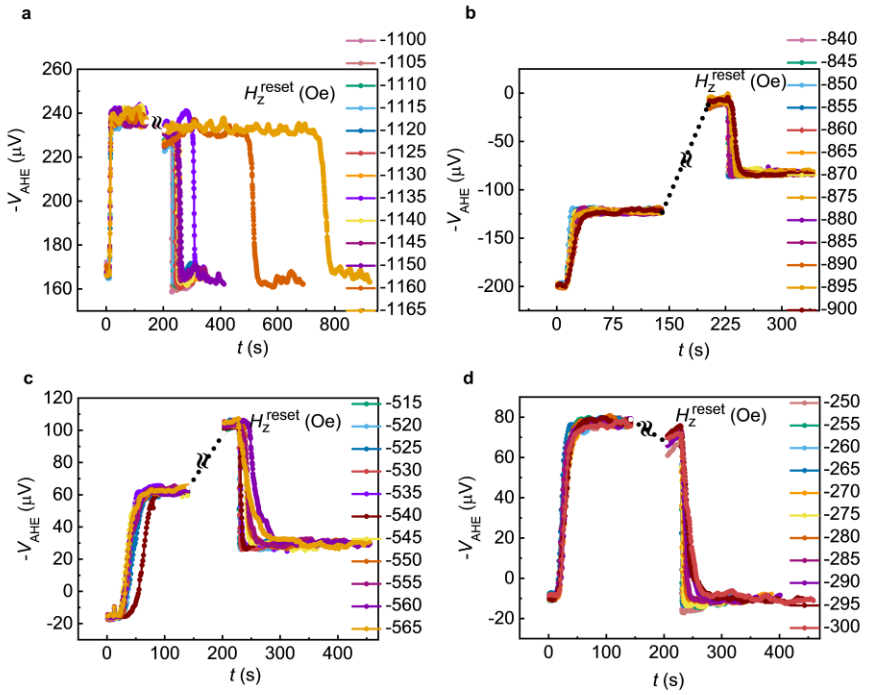

Fig. S19. Investigations of the return velocity of the domain walls which determine the time it takes for self-reset process. The AHE voltages used for estimating the return velocity with (a) H_{ex} = 1504 Oe, (c) H_{ex} = 1220 Oe, (d) H_{ex} = 885 Oe and (e) H_{ex} = 645 Oe.

Supplementary Information 2.4

For SOT driven Neuron:

- I_{write} = 15 mA (For SOT driven 8 neuron repetition, this is the applied I_{write})
- I_{read} = 100 µA
- Duration of I_{write} = 1 ms
- Duration of I_{read} = 100 ms

Power consumption, P = I^2R = $(J_{activation}\, w\, h_{Pt})^2 \frac{\rho l}{w\, h_{Pt}} = J_{activation}^2\, w\, h_{Pt} \rho l$

Here, $J_{activation}$ is the current density required for neuronal activation. R (1.9 kΩ), w (5 µm), $h_{Pt}\ (5\ nm), \rho\ (67\ \mu\Omega - \mathrm{cm}) and\ l\ (150\ \mu m)$ corresponds to resistance, width, thickness, resistivity and length of the Pt SOT layer respectively. $\boldsymbol{\rho}$ has been calculated using parallel circuit model[13].

So. P= $(2\times10^{11})^2 \times 5 \times 10^{-6} \times 5 \times 10^{-9} \times 67 \times 10^{-8} \times 150 \times 10^{-6}$ = 100.5 mW

For each cycle of neuron activation of the SOT driven 8 repetition cycle, we applied 20 pulses of 15 mA write current for integration. So, energy required for each cycle of neuron activation, E = (P × t_{write}) ×n = $100.5 \times10^{-3} \times 1 \times 10^{-3} \times 20$ = 2.01 mJ

Here, t_{write} is the write current pulse width, n is the pulse number for neuron activation in a single cycle.

Now, for read current the pulse amplitude was 100 µA and pulse width was 100 ms, so energy required for read current during one cycle neuron activation,

P' = $(100\times10^{-6})^2 \times 5 \times 10^{-6} \times 5 \times 10^{-9} \times 67 \times 10^{-8} \times 150 \times 10^{-6} = 2.51 \times 10^{-32}$ W

E' = = (P' × t_{read}) ×n = $2.51 \times 10^{-32} \times 100 \times 10^{-3} \times 20 = 50.2 \times 10^{-33}$ J

So, the energy required for neuron activation for read current is almost negligible and the write current energy per cycle of neuron activation operation is 2.01 mJ

The power consumption and energy required per cycle can be lowered by minimizing the device size to nm scale and taking the writing current pulse width in microsecond to nanosecond range.

- As for example If we estimate the power consumption by taking into account the device width as 180 nm and device length as 180 nm and compare with 180 nm technology node (energy/spike = 5 fJ) used neuron then the energy consumption for our neuron activation will be as P1 = $(2\times10^{11})^2 \times 180 \times 10^{-9} \times 5 \times 10^{-9} \times 67 \times 10^{-8} \times 180 \times 10^{-9}$ = 4.34 µW and the energy required for neuron activation = E1 = (P × t_{write}) ×n = $4.34 \times10^{-6} \times 1 \times 10^{-3} \times 20$ = 86.8 nW.
- As for another example If we estimate the power consumption by taking into account the device width as 55 nm and device length as 55 nm compared with 55 nm technology complementary metal–oxide–semiconductor process (energy/converting = 129.6 fJ , at a certain resolution of direct small-current converter) used neuron then the energy consumption for our neuron activation will be as P2 = $(2\times10^{11})^2 \times 55 \times 10^{-9} \times 5 \times 10^{-9} \times 67 \times 10^{-8} \times 55 \times 10^{-9}$ = 0.405 µW and the energy required for neuron activation = E2 = (P × t_{write}) ×n = $0.405 \times10^{-6} \times 1 \times 10^{-3} \times 20$ = 8.1 nW. By further optimizing the microsecond

range write current pulse width and current amplitude the energy consumption/neuron activation might be comparable with the literature values.

Supplementary information 2.5

LIF Neuron Model

The classical first-order differential equation of the LIF neuro model is expressed as:

$$\tau \frac{dV(t)}{dt} = -(V(t) - V_{reset}) + RI(t)$$

Where $V(t)$ is the membrane potential, V_{reset} is the resting potential, $I(t)$ is the input current, R is the membrane resistance.τ is the time constant. In the proposed model, this equation is modified to introduce separate leak and integration time constants τ_{leak} and $\tau_{integration}$. The discrete-time update equation used in the SNN architecture becomes:

$$V(t + \Delta t) = V(t) \left(1 - \frac{\Delta t}{\tau_{leak}}\right) + \frac{\Delta t}{\tau_{integration}} I(t)$$

Where Δt is the simulation step, τ_{leak} and $\tau_{integration}$ are time constants, and $I(t)$is the synaptic input current. A spike is generated when the membrane voltage exceeds the threshold,

$$S(t) = H\,(V(t) - V_{th})\,, \quad V(t) \leftarrow V_{th}\ if\ S(t) = 1$$

where H (·) denotes the Heaviside step function and S(t) is the spike output function of the LIF neuron. Since H (·) is non-differentiable, its derivative is zero almost everywhere and undefined at the threshold, which leads to the dead neuron problem, where gradients vanish and weights cannot be updated.

Input Encoding

The training of the spiking neural network (SNN) for MNIST digit recognition begins with each 28×28 grayscale image being flattened into a 784-dimensional vector, where each element represents a pixel intensity. These static pixel values are converted into temporal spike trains using Poisson encoding, a probabilistic method in which brighter pixels have a higher probability of generating spikes across a defined number of discrete time steps T. This encoding transforms the static image into a dynamic, spike-based representation suitable for processing by spiking neurons.

Synaptic Current and Neuron Dynamics

The input spike trains are fed into the network's fully connected layers. Each neuron in a hidden layer receives input spikes from all neurons in the previous layer through synaptic weights w_{ij}, which are initially randomly assigned. These weights determine the strength of influence each incoming spike has on the postsynaptic neuron. At each time step, the input current to a neuron is computed as the weighted sum of incoming spikes:

$$I_i(t) = \sum w_{ij} S_j(t)$$

Where, $S_j(t)$ is the spike (0 or 1) from presynaptic neuron j. This current drives the neuron's membrane potential, which evolves according to Leaky Integrate-and-Fire (LIF) dynamics, controlled by the integration constant ($\tau_{integration}$) and leak constant (τ_{leak}). When the membrane potential

exceeds a threshold, the neuron emits a spike and resets. This process continues for all time steps, allowing neurons to encode the input in a temporal spike pattern.

Regularization and Output Representation

During training, **dropout regularization** is applied to hidden layers, where a random subset of neurons is temporarily deactivated. This prevents the network from relying too heavily on specific neurons, encouraging distributed representations and reducing overfitting. At the output layer, spikes from the last hidden layer are integrated over all time steps. The total spike count for each output neuron is computed, and the neuron with the highest count represents the predicted digit. For example, if the output neuron corresponding to class '3' emits the most spikes, the network predicts the digit as '3'.

Learning and Optimization

Weight Update Rule

The network's prediction is compared with the true label using cross-entropy loss, which measures the discrepancy between the predicted and actual class distributions. Because spikes are binary and non-differentiable, standard gradient-based optimization cannot be applied directly. To overcome this, a surrogate gradient is used—a smooth approximation of the spike function derivative—allowing gradients to propagate through the network during backpropagation. The gradient of the loss with respect to each synaptic weight w_{ij} is calculated as:

$$\frac{\partial L}{\partial w_{ij}} = \sum_t \frac{\partial L}{\partial S_i(t)} \cdot \frac{\partial S_i(t)}{\partial V_i(t)} \cdot S_j(t)$$

Here, $\frac{\partial S_i(t)}{\partial V_i(t)}$ is the surrogate derivative, $\frac{\partial L}{\partial S_i(t)}$ is the error propagated from the output, and $S_j(t)$ is the presynaptic spike. Using an optimizer like SGD or Adam, synaptic weights are updated:

$$w_{ij} \leftarrow w_{ij} - \eta \cdot \frac{\partial L}{\partial w_{ij}}$$

This update strengthens connections that help generate correct output spikes and weakens others, progressively refining the network's ability to recognize digits.

Surrogate Gradient Approximation

Supervised training of the proposed LIF-SNN is performed on the MNIST and F-MNIST datasets using the surrogate gradient method to overcome the non-differentiability of spike generation:

$$\frac{\partial L}{\partial W} = \frac{\partial L}{\partial S} \cdot \frac{\partial S}{\partial U} \cdot \frac{\partial U}{\partial I} \cdot \frac{\partial I}{\partial W}$$

To address this, the surrogate gradient method replaces the non-differentiable step function with a smooth approximation during the backward pass while preserving the original threshold dynamics in the forward pass. For example, an arctan surrogate is used:

$$\tilde{S}(U) = \frac{1}{\pi} \arctan(\pi U), \quad \frac{d\tilde{S}}{dx} = \frac{1}{\pi} \frac{1}{1+(\pi U)^2}, \quad U = V(t) - V_{th}$$

More generally, the gradient is approximated as

$$\frac{\partial S(t)}{\partial U\ (t)} \approx \sigma'(V(t)\ -\ V_{th}\ )$$

where σ (·) may be sigmoid, fast-sigmoid, or arctan, this ensures that even non-firing neurons receive non-zero gradients, preventing them from becoming permanently inactive.

Backpropagation Through Time (BPTT) and Loss Function

Training proceeds via backpropagation-through-time (BPTT), which unfolds the recurrent membrane dynamics across simulation steps. At each step, LIF updates govern the forward dynamics, while surrogate gradients propagate error signals during the backward pass. At the output layer, spikes are accumulated into class-specific spike counts:

$$z_c = \sum_{t=1}^{T} S_c(t)$$

which were treated as logits for classification. The training objective was the cross-entropy loss,

$$L = \ -\log \frac{e^{z_y}}{\sum_j e^{z_j}}$$

where z_y is the spike count of the correct class. This formulation enables the direct application of standard classification objectives to discrete spike outputs.

Optimization and Stabilization

Optimization was performed using the Adam optimizer ($lr = 10^{-3}$) with weight decay (10^{-5})) to mitigate overfitting. To stabilize training, dropout (p=0.2), Gaussian input noise (σ=0.01), and gradient clipping (max-norm = 1.0) were employed. These techniques enhanced robustness and generalization across both datasets.

Testing Phase

Once training is completed, the spiking neural network (SNN) is evaluated on unseen test samples in a forward-only manner, with synaptic weights frozen. The inference procedure mirrors the forward dynamics of training but excludes gradient computations, surrogate derivatives, and weight updates.

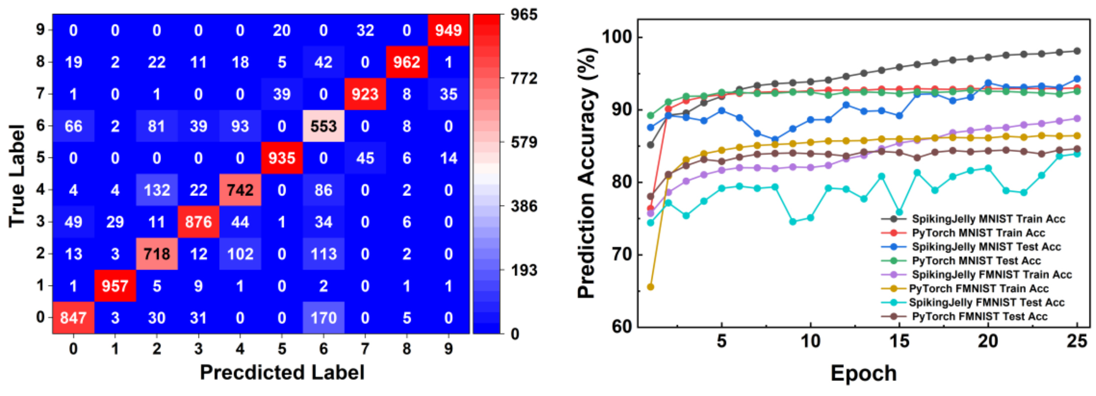

(a) (b)

Fig. S20. (a) The confusion matrix of the F-MNIST. (b) The training and testing accuracies of the proposed SNN architecture are evaluated on the PyTorch and SpikingJelly frameworks for both the MNIST and F-MNIST datasets, with the integration and leak time constants fixed at 3 ms.

Input Encoding:

Each test image from MNIST or Fashion-MNIST is first flattened into a 784-dimensional vector and converted into temporal spike trains using the same Poisson rate-coding scheme as in training. For a pixel with normalized intensity P_j∈[0,1], the spike train is generated as:

$$S_j(t) \sim Bernoulli\ (p_i), t = 1,2, \dots, T,$$

where brighter pixels produce spikes with higher probability. To mitigate stochastic variations in encoding, predictions may be averaged across multiple Poisson trials, though single-run inference is also commonly reported.

Output Representation:

As spikes propagate through the hidden layers, the output layer accumulates spikes over all time steps. For class ccc, the accumulated spike count is:

$$z_c = \sum_{t=1}^{T} S_c(t)$$

The predicted label corresponds to the class with the maximum spike count:

$$\tilde{y} = argmax\ z_c$$

These spike counts can also be normalized using a softmax function to obtain probability-like outputs if required.

Performance Evaluation:

Predictions are compared against ground truth labels to compute classification accuracy. Evaluation metrics typically include:

Overall accuracy: fraction of correctly classified samples.

Confusion matrix: detailed distribution of correct vs. misclassified examples.

Table S5: The operational flexibility under variations of the $\tau_{\text{integration}}$ and τ_{leak} across a wide temporal range, from 3 ms down to 3 μs.

τ_{Leak} / τ_{Int}	$3e^{-3}$	$3e^{-4}$	$3e^{-5}$	$3e^{-6}$
$3e^{-3}$	**92.57**	**91.86**	**84.21**	**32.87**
$3e^{-4}$	**92.18**	**92.02**	**92.43**	**22.97**
$3e^{-5}$	**89.13**	**87.83**	**91.99**	**10.69**
$3e^{-6}$	**94.09**	**95.23**	**84.94**	**28.38**

Sigmoid surrogate function as the surrogate gradient: we also incorporated the sigmoid surrogate function as the surrogate gradient and examined its effect on both the training convergence speed and the test accuracy of the proposed SNN model. For a fair comparison, all simulation parameters were kept identical, with the surrogate gradient function being the only variable. The resulting performance is shown in the figure, which presents the training and testing accuracies of the SNN over 25 epochs. Using the sigmoid surrogate function, the model achieves 97.47% training accuracy and 95.92% testing accuracy on MNIST, and 85.68% training accuracy and 78.58% testing accuracy on F-MNIST, with the integration and leak time constants fixed at 3 ms.

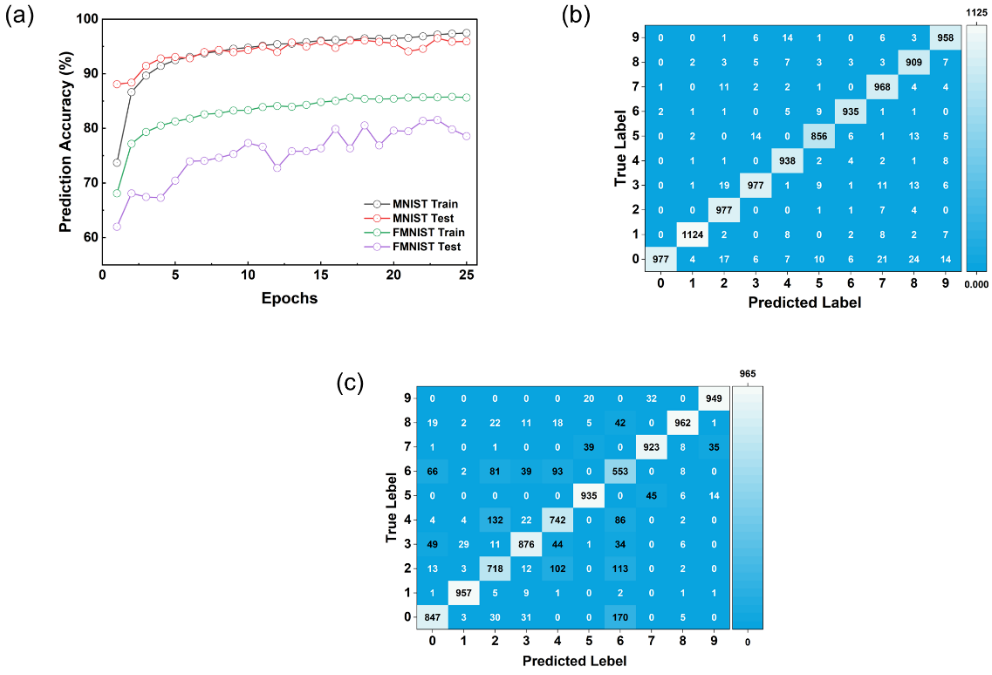

Fig.S21. (a) Training and testing accuracy curves for the classification of MNIST and F-MNIST datasets using sigmoid surrogate function. A detailed visualization of classification performance by comparing predicted labels against the True labels of the classes for the (b) for the MNIST and (c) F-MNIST datasets.

References

1. Trabzon, A. B., Goto, T., & Onbasli, M. C. (2025). Comprehensive Micromagnetic Modeling: Practical Techniques, Applications, and Emerging Challenges. IEEE Access.

2. Sinova, J., Valenzuela, S. O., Wunderlich, J., Back, C. H., & Jungwirth, T. (2015). Spin hall effects. Reviews of modern physics, 87(4), 1213-1260.

3. Vansteenkiste, A., Leliaert, J., Dvornik, M., Helsen, M., Garcia-Sanchez, F., & Van Waeyenberge, B. (2014). The design and verification of MuMax3. AIP advances, 4(10).

4. Rahaman, Hasibur, Durgesh Kumar, Hong Jing Chung, Ramu Maddu, Sze Ter Lim, Tianli Jin, and S. N. Piramanayagam. 'Diode characteristics in magnetic domain wall devices via geometrical pinning for neuromorphic computing.' ACS Applied Materials & Interfaces 15, no. 12 (2023): 15832-15838.

5. Xie, X. *et al.* Engineering Spin Configurations of Synthetic Antiferromagnet by Controlling Long-Range Oscillatory Interlayer Coupling and Neighboring Ferrimagnetic Coupling. *Adv. Mater.* **35**, 2208275 (2023).

6. Prudnikov, A., Li, M., De Graef, M. & Sokalski, V. Simultaneous Control of Interlayer Exchange Coupling and the Interfacial Dzyaloshinskii–Moriya Interaction in Ru-Based Synthetic Antiferromagnets. *IEEE Magn. Lett.* **10**, 1–4 (2019).

7. Kossak, A. E., Huang, M., Reddy, P., Wolf, D. & Beach, G. S. D. Voltage control of magnetic order in RKKY coupled multilayers. *Sci. Adv.* **9**, eadd0548 (2023).

8. Krishnia, S. *et al.* Role of RKKY torque on domain wall motion in synthetic antiferromagnetic nanowires with opposite spin Hall angles. *Sci. Rep.* **7**, 11715 (2017).

9. Pai, C. F., Mann, M., Tan, A. J., & Beach, G. S. (2016). Determination of spin torque efficiencies in heterostructures with perpendicular magnetic anisotropy. Physical Review B, 93(14), 144409.

10. Parkin, S. S., Hayashi, M., & Thomas, L. (2008). Magnetic domain-wall racetrack memory. science, 320(5873), 190-194.

11. Hayashi, M., Thomas, L., Rettner, C., Moriya, R., Jiang, X., & Parkin, S. S. (2006). Dependence of Current and Field Driven Depinning of Domain Walls on Their Structure<? format?> and Chirality in Permalloy Nanowires. Physical review letters, 97(20), 207205.

12. Lim, G. J., Gan, W. L., Law, W. C., Murapaka, C., & Lew, W. S. (2020). Spin-orbit torque induced multi-state magnetization switching in Co/Pt hall cross structures at elevated temperatures. Journal of Magnetism and Magnetic Materials, 514, 167201.

13. Bhatti, S., Das, S., Shaik, A., Chan, J., Kay, Y. S., Li, S., & Piramanayagam, S. N. (2024). Enhancing damping-like efficiency by low-energy mixed ions bombardment. Applied Physics Letters, 124(2).
